# Toward Sustainable Li–S Battery Using Scalable Cathode and Safe Glyme-Based Electrolyte

**DOI:** 10.1021/acsaem.3c01966

**Published:** 2023-11-08

**Authors:** Vittorio Marangon, Edoardo Barcaro, Eugenio Scaduti, Filippo Adami, Francesco Bonaccorso, Vittorio Pellegrini, Jusef Hassoun

**Affiliations:** † Graphene Laboratories, Istituto Italiano di Tecnologia, Via Morego 30, Genoa 16163, Italy; ‡ Department of Chemical, Pharmaceutical and Agricultural Sciences, University of Ferrara, Via Fossato di Mortara 17, Ferrara 44121, Italy; § BeDimensional S.p.A., Lungotorrente Secca 30R, Genova 16163, Italy; ∥ National Interuniversity Consortium of Materials Science and Technology (INSTM), University of Ferrara Research Unit, Via Fossato di Mortara, 17, Ferrara 44121, Italy

**Keywords:** Li–S battery, glyme electrolyte, low flammability, MWCNTs, current collector, E/S ratio

## Abstract

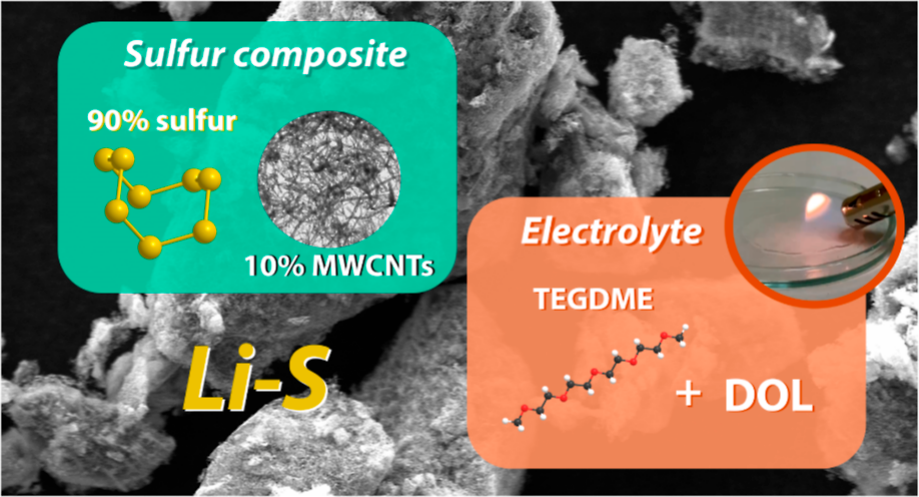

The search for safe electrolytes to promote the application of lithium–sulfur (Li–S) batteries may be supported by the investigation of viscous glyme solvents. Hence, electrolytes using nonflammable tetraethylene glycol dimethyl ether added by lowly viscous 1,3-dioxolane (DOL) are herein thoroughly investigated for sustainable Li–S cells. The electrolytes are characterized by low flammability, a thermal stability of ∼200 °C, ionic conductivity exceeding 10^–3^ S cm^–1^ at 25 °C, a Li^+^ transference number of ∼0.5, electrochemical stability window from 0 to ∼4.4 V vs Li^+^/Li, and a Li stripping-deposition overpotential of ∼0.02 V. The progressive increase of the DOL content from 5 to 15 wt % raises the activation energy for Li^+^ motion, lowers the transference number, slightly limits the anodic stability, and decreases the Li/electrolyte resistance. The electrolytes are used in Li–S cells with a composite consisting of sulfur and multiwalled carbon nanotubes mixed in the 90:10 weight ratio, exploiting an optimized current collector. The cathode is preliminarily studied in terms of structure, thermal behavior, and morphology and exploited in a cell using standard electrolyte. This cell performs over 200 cycles, with sulfur loading increased to 5.2 mg cm^–2^ and the electrolyte/sulfur (E/S) ratio decreased to 6 μL mg^–1^. The above sulfur cathode and the glyme-based electrolytes are subsequently combined in safe Li–S batteries, which exhibit cycle life and delivered capacity relevantly influenced by the DOL content within the studied concentration range.

## 1 Introduction

Li–S battery is certainly the most appealing postlithium-ion system of choice for powering the next generation of electric devices due to its relevant energy density which can exceed 450 W h kg^–1^.^1−4^ The present-state Li–S cell involves electrolytes based on solution of various salts in dioxolane (DOL) and dimethoxyethane (DME), characterized by high ionic conductivity and Li^+^ transference number.^5,6^ Despite these favorable characteristics, DOL:DME solutions have relevant flammability and can favor dendrite growth at the Li metal surface, as well as excessive mobility and reactivity of the lithium polysulfides formed during the electrochemical process, thus posing serious concerns on the Li–S cell safety and large-scale diffusion.^7^ Therefore, the development of new electrolyte media has been recognized as a priority in the optimization of the Li–S cell,^8^ and glymes with *n* ≥ 2 in the −(CH_2_CH_2_O)_*n*_– molecular formula have been indicated as the most suitable solvents for alternative and safe solutions.^9^ Promising electrolyte formulations have been proposed throughout the years by using various concentrations of conductive salts, cosolvents, and polysulfides in either diethylene glycol dimethyl ether (DEGDME),^10−13^ triethylene glycol dimethyl ether (TREGDME),^14,15^ or tetraethylene glycol dimethyl ether (TEGDME),^16,17^ as well as in solid configurations exploiting polyethylene glycol dimethyl ether with high molecular weight.^18,19^ Among the liquid solvents, TREGDME and TEGDME appeared appealing since they have particularly low volatility and flammability and still moderate viscosity for allowing sufficient charge transport at the room temperature.^20^ In addition, the glyme-based solutions have been indicated to limit the depletion of the electrolyte at the lithium electrode surface and promote homogeneous lithium deposition, thus providing a regular electrode/electrolyte interphase and mitigating the formation of lithium dendrites. This favorable process has been attributed to the relatively long ether chain of glymes that improves the stability toward radical species formed during the Li–S conversion process and, at the same time, enhances the lithium transport across the electrolyte bulk through the chelation of the Li^+^ ions by the glyme molecules allowing an efficient interchain hopping mechanism.^9^ Further steps toward practical configurations of the Li–S battery are the increase of active material in the sulfur cathode for achieving adequate gravimetric energy density^21,22^ and the limitation of electrode thickness and of the electrolyte volume to get a satisfactory volumetric energy density.^23,24^ In this regard, thin-layer current collectors suitable for Li–S battery application have been recently developed by coating aluminum with various carbons, including multiwall carbon nanotubes (MWCNTs), graphene flakes formed by limited number of layers, and amorphous substrates.^25,26^ These new supports have been used as the current collectors in Li–S cells achieving volumetric energy density comparable to Li-ion ones (i.e., 500 W h L^–1^) and much higher gravimetric energy (480 W h kg^–1^).^27^ In these cells, the use of the most diffused electrolyte based on DOL:DME, LiTFSI salt, and LiNO_3_ additive was adopted to form a protective film on the lithium metal surface and limit the direct reaction of the dissolved polysulfides (particularly Li_2_S_8_) with the alkali metal at the anode side (i.e., the shuttle reaction).^27^ Despite this strategy appeared suitable for boosting the performance, the cells were still affected by issues ascribed to the flammable nature of the electrolyte and the formation of dendrites at the metal surface, with intrinsic safety limit.^28,29^ This drawback was relevant in view of a large-scale diffusion of the Li–S battery, in particular in the electric vehicle (EV) field which requires challenging safety standards.^7,30,31^ Therefore, we exploited in this work the low flammability of TEGDME-based solutions and a sulfur-MWCNT composite including 90 wt % of active material cast on a low-thickness MWCNT-coated Al support to obtain Li–S cells characterized both by suitable energy density and remarkable safety content. It is worth mentioning that high sulfur loading has also been recently achieved by replacing the conductive carbon matrix of the sulfur composite with metal or oxide particles,^32,33^ which is a consolidated strategy to achieve fast kinetics also employed in other devices as demonstrated in literature works.^34−36^ Additional strategies to improve the cathode performance may rely on optimized configurations including modified sulfurized polyacrylonitrile (SPAN)^37^ and multilayered structures exploiting synergic cathode chemistries.^38,39^ The already known high viscosity of TEGDME (3.3–3.7 mPa s)^9,40^ may represent a non-negligible limit hindering the thin-layer cell application, particularly in view of the relatively low wettability of the laminated sulfur electrode using carbon-coated Al-support.^41^ This relevant issue can be mitigated by preparing binary solutions of TEGDME and a cosolvent with lower viscosity such as DOL (0.6 mPa s),^42^ hence allowing cell operation with limited charge-transfer resistance.^43^ Herein, we have proposed electrolytes predominantly including TEGDME with a limited fraction of DOL, i.e., either 5, 10, or 15 wt %. The main advantage of the TEGDME-/DOL-based electrolyte compared to that of the DME-/DOL-based one is the negligible flammability of the former rather than the high flammability of the latter. This aspect represents a very important factor for allowing diffusion of the Li–S battery. Furthermore, the combination of TEGDME and DOL is expected to reciprocally compensate the respective drawback of the two solvents, that is, the high viscosity of the former and the relevant flammability and low stability toward the Li_2_S_*x*_ intermediates of the latter. Indeed, the TEGDME/DOL solutions can rely on the high chemical stability of the glyme molecule and advantageously exploit the low viscosity and the film-forming ability of the cyclic ether,^44^ thus providing a suitable and safe environment for proper operation of the Li–S cell. The electrolytes have been investigated in terms of conductivity and interphase chemical stability by using electrochemical impedance spectroscopy (EIS). Furthermore, the electrochemical stability window was determined by cyclic voltammetry (CV) and linear scan voltammetry (LSV), the lithium transference number by chronoamperometry and EIS, and the thermal behavior through thermogravimetric analysis (TGA). At the same time, structure, morphology, and thermal stability of the sulfur composite have been determined via X-ray diffraction (XRD), electron microscopy, and TGA, respectively, before being used for cathode preparation. The sulfur electrode has been preliminarily cycled in a Li cell using a DOL:DME-control electrolyte, prior to testing alongside the glyme-based solutions in Li–S batteries. Therefore, our novel approach provides the full characterization in parallel of a nonflammable electrolyte and of a sulfur cathode prepared with facile synthesis pathways including environmentally friendly materials and their application in a safe Li–S cell. The results of this study can actually promote the development at the large scale of Li–S batteries with enhanced performances and low economic impact due to the limited cost of sulfur and glymes compared to that of the electrode and electrolyte typically employed in the Li-ion batteries.^45,46^

## 2 Experimental Section

### 2.1 Glyme Electrolyte Preparation and Characterization

The precursor electrolyte was prepared in an Ar-filled glovebox (MBraun, H_2_O and O_2_ below 1 ppm) by dissolving lithium bis(trifluoromethanesulfonyl)imide [LiTFSI, LiN(SO_2_)_2_(CF_3_)_2_, 99.95% trace metal basis, Sigma-Aldrich] and lithium nitrate (LiNO_3_, 99.99% trace metal basis, Sigma-Aldrich) in TEGDME [CH_3_(OCH_2_CH_2_)_4_OCH_3_, ≥ 99%, Sigma-Aldrich] with a concentration of 1 mol kg_solvent_^–1^ for each salt. Subsequently, three solutions were obtained by adding 1,3-dioxolane (DOL, anhydrous, contains ca. 75 ppm of BHT as the inhibitor, 99.8%, Sigma-Aldrich) in various concentrations to the precursor electrolyte, that is, 5, 10, and 15 wt % with respect to the mass of the initial solution. The electrolytes are indicated in the text as TE-5%, TE-10%, and TE-15%, respectively, and Table 1 summarizes acronyms and the corresponding compositions.

**Table 1:** Electrolyte Acronyms and the Corresponding Compositions

electrolyte acronym	composition
TE-5%	TEGDME, 1 mol kg^–^^1^ LiTFSI, 1 mol kg^–^^1^ LiNO_3_ + 5% DOL
TE-10%	TEGDME, 1 mol kg^–^^1^ LiTFSI, 1 mol kg^–^^1^ LiNO_3_ + 10% DOL
TE-15%	TEGDME, 1 mol kg^–^^1^ LiTFSI, 1 mol kg^–^^1^ LiNO_3_ + 15% DOL

Prior to use, a Karl Fischer 899 Coulometer (Metrohm) was employed to verify the water content below 10 ppm of TEGDME and DOL, which was achieved upon prolonged storage at room temperature of the solvents with molecular sieves (rods, 3 Å, size 1/16 in., Honeywell Fluka) previously dried under vacuum at 280 °C for 5 days, while LiTFSI and LiNO_3_ were dried under vacuum for 2 days at 110 °C. TGA of the electrolytes was performed through a Mettler-Toledo TGA 2 instrument by running temperature scans in the 25–800 °C range at 5 °C min^–1^ with a N_2_ flow of 50 mL min^–1^. Fourier transform infrared (FT-IR) spectra of the solutions were recorded via a Bruker Vertex V70 instrument set up in the transmittance mode. Electrochemical measurements were carried out on either CR2032 coin-type cells (MTI Corp.) using electrodes with 14 mm diameter or T-type Swagelok cells using electrodes with 10 mm diameter assembled in an Ar-filled glovebox. The ionic conductivity of the solutions was evaluated by running EIS measurements at various temperatures on stainless-steel|electrolyte|stainless-steel symmetrical coin cells where the solution was held by an O-ring (23–5FEP-2–50, CS Hyde) with an internal diameter of 10 mm. The O-ring thickness of 127 μm allowed us to fix the cell constant at 0.016 cm^–1^. The EIS spectra were recorded in the 500 kHz–100 Hz frequency range using an alternate voltage signal of 10 mV, while the cell temperature was controlled by a Julabo F12 instrument. The ionic conductivity data were used to calculate the activation energy of the electrolytes via the Arrhenius equation (eq 1)^47^
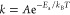
1where *k* is the slope of the Arrhenius plot, *A* is a pre-exponential factor, *E*_a_ is the activation energy for ion motion (eV), *k*_B_ is the Boltzmann constant (8.52 × 10^–5^ eV K^–1^), and *T* is the temperature (K). The Li^+^ transference number (*t*^+^) of the electrolytes was estimated through the Bruce–Vincent–Evans method^48^ using Li|Li symmetrical cells where the 14 mm-diameter Li electrodes were separated by two glass-fiber Whatman GF/B 16 mm-diameter discs. Accordingly, the cells were subjected to chronoamperometry tests by applying a potential signal (Δ*V*) of 30 mV for 90 min, while Nyquist plots were recorded via EIS before and after polarization in the 500 kHz–100 mHz frequency range using a 10 mV alternate voltage signal. The chronoamperometry and EIS outcomes were used in eq 2(48)
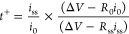
2where *i*_0_ and *i*_ss_ are the current values at the initial and steady state, respectively, and *R*_0_ and *R*_ss_ are the interphase resistance values before and after cell polarization, respectively (see the resistance evaluation method below). Lithium stripping-deposition tests were performed exploiting a current of 0.1 mA cm^–2^ for galvanostatic charge/discharge processes of Li|Li symmetrical T-cells where one glass-fiber Whatman GF/B 10 mm-diameter disc separated the Li electrodes. The electrolyte stability upon aging was investigated on Li|Li symmetrical cells with one glass-fiber Whatman GF/A 16 mm-diameter disc as the separator by running EIS between 500 kHz and 100 mHz with a 10 mV alternate voltage signal every 2 h for the first 14 h after assembly and subsequently on daily basis for 18 days. The electrochemical stability window (ESW) of the solutions was determined using Li cells that employed a Super P carbon (SPC, Timcal)-based working electrode separated from the 14 mm-diameter lithium anode by a glass-fiber Whatman GF/A 16 mm-diameter disc. The SPC-based cathodes were prepared by dispersing SPC (80 wt %) and poly(vinylidene fluoride) (20 wt %, PVDF, Solef 6020)-binding polymer in *N*-methyl-2-pyrrolidone (NMP, Sigma-Aldrich) to obtain a viscous slurry, which was cast on either Al or Cu foils with the aid of a doctor blade tool (MTI Corp.). The electrode tapes were dried on a hot plate at 70 °C for 3 h and cut into 14 mm-diameter discs, which were dried under vacuum at 110 °C for 3 h before being transferred in an Ar-filled glovebox. Anodic and cathodic regions of the ESW were investigated by performing either LSV on Li|SPC-Al cells from the open-circuit voltage (OCV) condition to 5 V vs Li^+^/Li or CV between 0.01 and 2.0 V vs Li^+^/Li on Li|SPC-Cu cells. Both LSV and CV data were acquired at a 0.1 mV s^–1^ scan rate. All Nyquist plots obtained by EIS were fitted via nonlinear least-squares (NLLS) method with the Boukamp software.^49,50^ The NLLS analyses allowed us to describe the Li cell through an equivalent circuit composed of resistive (*R*) and constant-phase (*Q*) capacitive elements. In particular, the high-frequency intercept of the plot with the real axis is associated with the electrolyte resistance (*R*_e_); the amplitude of the high-medium frequency semicircle measures the interphase resistance *R*_i_ which includes contributes of the passivation film and charge transfers and is arranged in parallel with the *Q*_i_ capacitance in the (*R*_i_*Q*_i_) element, while the low-frequency semicircles (*R*_w_*Q*_w_) or tilted lines (*Q*_w_), respectively, represent either the finite-length or semiinfinite Warburg-type Li^+^ diffusion.^49−51^ Only fitting results with a χ^2^ value of the order of 10^–4^ or lower were considered suitable. Voltammetry and EIS measurements were performed by using a VersaSTAT MC Princeton Applied Research (PAR-AMETEK) instrument, while the galvanostatic cycling data were recorded through a MACCOR series 4000 battery test system.

### 2.2 Sulfur Composite Synthesis and Characterization

Elemental sulfur (≥99.5%, Riedel-de Haën) and MWCNTs (>90% carbon basis, *D* × *L*: 110–170 nm × 5–9 μm, Sigma-Aldrich) were mixed in the 90:10 w/w ratio and heated at 125 °C under magnetic stirring with a silicon oil bath until melting of sulfur and uniform mixing with MWCNTs. The viscose mixture was subsequently quenched at room temperature until solidification and ground in an agate mortar to obtain a fine powder. The composite is indicated in the text as S:MWCNTs 90:10 w/w. XRD patterns of S:MWCNTs 90:10 w/w and bare MWCNTs were acquired using a Bruker D8 Advance equipped with a Cu Kα source (8.05 keV) by performing scans over the 10–90° 2θ range with a step size of 0.02° and a rate of 10 s step^–1^. TGA was performed via a Mettler-Toledo TGA 2 instrument between 25 and 1000 °C under a N_2_ flow of 50 mL min^–1^ at 5 °C min^–1^. Scanning electron microscopy (SEM) images were captured by a Zeiss EVO 40 microscope using a LaB_6_ thermionic electron gun in both secondary electrons and backscattered electrons mode. Energy-dispersive spectroscopy (EDS) elemental maps were recorded on the SEM backscattered electrons images through a X-ACT Cambridge Instrument associated with the microscope. Transmission electron microscopy (TEM) images were acquired with a Zeiss EM 910 microscope equipped with a tungsten thermoionic electron gun working at 100 kV.

### 2.3 Li–S Cell Electrochemical Tests

The cathode current collector was prepared following the pathway reported in a previous work.^25^ Accordingly, a slurry composed by 90 wt % MWCNTs and 10 wt % PVDF dispersed in NMP was cast on a bare aluminum foil (thickness of 15 μm, MTI Corp.) with the aid of a doctor blade tool (MTI Corp.). The cathodic support was dried at room temperature, and the final MWCNTs loading was ∼1.3 mg cm^–2^. The sulfur electrodes were prepared by using the MWCNTs-coated aluminum support. The electrode was obtained via doctor blade casting of a slurry composed by 80% S:MWCNTs 90:10 w/w, 10% poly(vinylidene fluoride-*co*-hexafluoropropylene) (PVDF-HFP, Kynar Flex 2801) as the polymer binder, and 10% few-layer graphene (produced through the WJM method, BeDimensional S.p.A.)^52^ as conductive carbon dispersed in tetrahydrofuran (THF,Sigma-Aldrich) through 1 h of magnetic stirring. The electrode tape was dried at room temperature, calendared with a MSK-2150 rolling machine (MTI Corp.) with final thickness from about 90 μm to about 140 μm, cut into discs with a diameter of 14 mm (geometrical area: 1.54 cm^2^), and dried overnight at 30 °C under vacuum before being transferred in an Ar-filled glovebox. The final sulfur loading on the electrodes ranged between 1.7 and 5.2 mg cm^–2^. CR2032 coin-type cells (MTI Corp.) were assembled in an Ar atmosphere by stacking a 14 mm-diameter lithium disc, an 18 mm-diameter Celgard 2400 separator soaked with the electrolyte (see volume below), and a sulfur cathode. A control electrolyte was prepared by mixing DOL and 1,2-dimethoxyethane (DME, anhydrous, 99.5%, inhibitor-free, Sigma-Aldrich) solvents in the 1:1 w/w ratio and dissolving LiTFSI and LiNO_3_ both in a concentration of 1 mol kg_solvent_^–1^. Analogously to the other solvents, DME water content lower than 10 ppm was achieved via prolonged storage under dry molecular sieves (rods, 3 Å, size 1/16 in., Honeywell Fluka) and confirmed prior to use by a Karl Fischer 899 Coulometer (Metrohm). The sulfur electrode was initially tested in a Li cell using the DOL:DME-control electrolyte. In particular, galvanostatic cycling measurements were performed through constant current rates of either C/5 or C/3 (1C = 1675 mA g^–1^) between 1.7 and 2.8 V for a sulfur loading of 2.2–2.3 mg cm^–2^ using an E/S ratio of 10 μL mg^–1^, while cathodes with a sulfur loading of 5.2 mg cm^–2^ were employed for galvanostatic tests at C/10 in the 1.7–2.8 V voltage range using an E/S ratio of 6 μL mg^–1^. Rate capability tests were also carried out exploiting a sulfur loading of 2.2 mg cm^–2^ and an E/S ratio of 10 μL mg^–1^ by increasing current rate every 5 cycles from C/10 to C/8, C/5, C/3, and C/2 and decreasing back to C/10 after 25 cycles. A voltage range from 1.8 to 2.8 V was used from C/10 to C/3, while limits of 1.7 and 2.8 V were exploited for C/2. CV tests were performed via potential scans between 1.8 and 2.8 V vs Li^+^/Li at a scan rate of 0.1 mV s^–1^ through a VersaSTAT MC Princeton Applied Research (PAR-AMETEK) instrument. The S:MWCNTs 90:10 w/w cathode was subsequently tested in a Li cell using the TE-5%, TE-10%, and TE-15% electrolytes via galvanostatic cycling measurements at C/5 constant rate (sulfur loading: 1.7–2.1 mg cm^–2^ and E/S ratio: 15 μL mg^–1^) between 1.7 and 2.8 V. Rate capability measurements were also performed at increasing rates of C/20, C/10, C/8, C/5, C/3, and C/2 before decreasing the current back at C/20 after 30 cycles (sulfur loading: ∼2.0 mg cm^–2^ and E/S ratio: 15 μL mg^–1^). The tests were carried out between 1.8 and 2.8 V from C/20 to C/8 and between 1.7 and 2.8 V from C/5 to C/2. The galvanostatic cycling measurements were all carried out with a MACCOR series 4000 battery test system.

## 3 Results and Discussion

**Figure 1:**
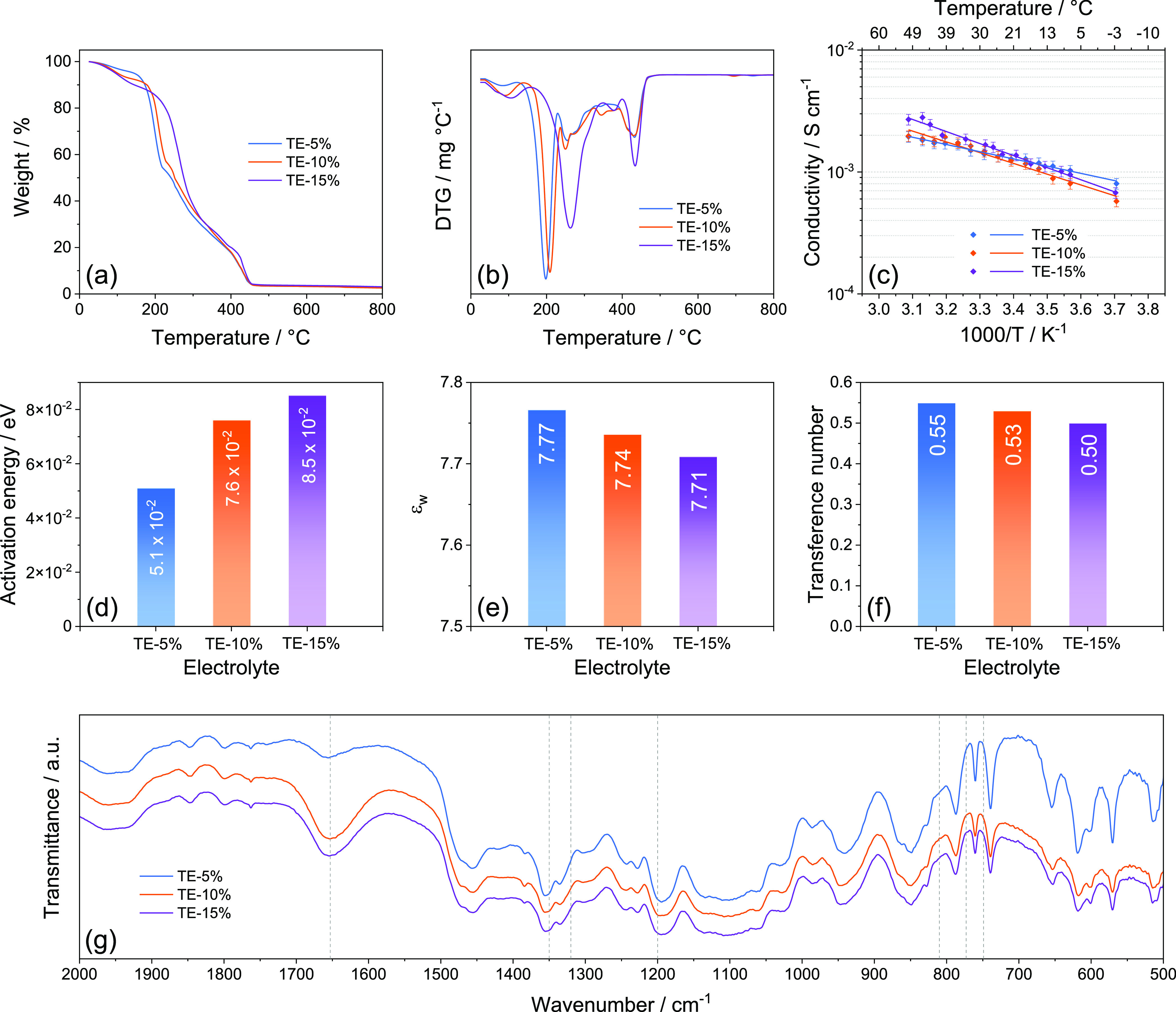
(a) TGA and (b) the corresponding DTG curves of the electrolytes acquired under N_2_ flow between 25 and 800 °C at the 5 °C min^–1^ rate; (c) ionic conductivity trends of the electrolytes reporting, in addition, the linear fit for each electrolyte; see Nyquist plots in Figure S1a–c in the Supporting Information; (d) histogram representation of the activation energy values calculated using the Arrhenius equation (eq 1) on the ionic conductivity trends in (c); (e) histogram representation of the weighted average dielectric constants (ε_w_) calculated considering the electrolyte solvents ratio and the ε values of pure DOL (7.1) and TEGDME (7.8); (f) histogram representation of the Li^+^ transference number (t^+^) of the electrolytes determined through the Bruce–Vincent–Evans method (eq 2); see chronoamperometric curves and related Nyquist plots in Figure S1d–f in the Supporting Information; (g) FT-IR spectra of the TE-5%, TE-10%, and TE-15% solutions. See Table 1 for electrolyte acronyms.

The safety content of the TE-5%, TE-10%, and TE-15% electrolytes (see Table 1 for compositions) is investigated through flammability tests in Movies S1, S2, and S3, respectively. Notably, none of the solutions presents ignition processes after direct flame exposure for 5 s, thus confirming the enhanced safety with respect to the conventional DOL:DME-control electrolyte, which shows instead immediate fire development, as revealed by Movie S4. The physical–chemical properties of the TE-5%, TE-10%, and TE-15% electrolytes are evaluated in Figure 1. The TGA (Figure 1a) and corresponding differential curves (DTG, Figure 1b) show for all electrolytes a first weight decrease at 150 °C with intensity growing alongside DOL concentration, likely due to the partial volatilization of the cyclic ether.^53^ The evaporation of TEGDME solvent takes place around 200 °C;^53^ however, the related weight loss is centered at 197 °C for TE-5%, 210 °C for TE-10%, and 263 °C for TE-15%. These discrepancies can be ascribed to the modification of the solvation environment caused by the increasing concentration of DOL, which may lead to formation of a cosolvent and influence the structure, as well as thermal behavior, of the solvent–salt complexes within the solution.^54^ The DTG curves further support the formation of specific salt–solvent complexes depending on the DOL content, in view of the differences observed in the multipeak profile between 250 and 400 °C, associated with weight losses due to solvent removal from crystallized-salt structures. In addition, the thermal analysis reveals at about 430 °C the weight decrease ascribed to LiTFSI degradation.^55^ It is worth noting that residual weight exhibited by the electrolytes at the end of the test can be attributed to LiNO_3_.^55^ A relatively lower impact of DOL concentration is observed in the conductivity plots reported in Figure 1c obtained from the EIS spectra displayed in Figure S1a–c in the Supporting Information. Indeed, TE-15% shows conductivity approaching 3 × 10^–3^ S cm^–1^ around 50 °C exceeding the one related to TE-5% and TE-10% of 2 × 10^–3^ S cm^–1^, while similar values are observed at room temperature (∼25 °C) near 1.5 × 10^–3^ S cm^–1^. Relevantly, at temperature as low as −3 °C, the TE-5%, TE-10%, and TE-15% solutions still exhibit conductivities of 8 × 10^–4^, 6 × 10^–4^, and 7 × 10^–4^ S cm^–1^, that is, suitable values to promote efficient operation in Li batteries. Furthermore, all the solutions show a linear conductivity trend with slope increasing in concomitance with the DOL concentration raise. The Arrhenius equation (eq 1) allows the estimation of the activation energy (*E*_a_), which identifies the energy barrier limiting the Li^+^ diffusion in the electrolyte.^47^ The calculated values are represented in the histogram of Figure 1d, which reveals the increase of *E*_a_ from TE-5% (5.1 × 10^–2^ eV) to TE-10% (7.6 × 10^–2^ eV) and TE-15% (8.5 × 10^–2^ eV). The increase of *E*_a_ by raising the content of the DOL in the solvent mixture may in part contrast with the expected viscosity and ion friction decreases that can, in principle, increase the Li^+^ mobility. On the other hand, this behavior may be justified by the variation of dielectric constant (ε) of the mixture due to the lower ε for DOL (7.1)^56^ compared to TEGDME (7.8),^57^ the chain-structure of which can efficiently promote the ion-pair dissociation and mobility due to the relevant content of oxygen atoms suitable for the Li^+^ coordination into Li–glyme complexes.^54^ Hence, the weighted average dielectric constant (ε_w_) of the solvent mixtures, calculated in Figure 1e considering the TEGDME:DOL ratio, decreases from 7.77 for TE-5% to 7.74 for TE-10% and to 7.71 for TE-15%. This trend agrees with that of *E*_a_ discussed previously, evidencing the complex influence of DOL on the glyme-based electrolyte properties, although the reduction of viscosity can increase the conductivity at the higher temperatures as observed herein. The Li^+^ transport features of the solutions are subsequently evaluated in a Li symmetrical cell for estimation of the Li^+^ transference number (t^+^) using the Bruce–Vincent–Evans equation (eq 2).^48^ The obtained values are displayed in Figure 1f as histogram columns, while Table 2 summarizes the parameters used for calculation obtained from the chronoamperometric curves and Nyquist plots collected in Figure S1d–f in the Supporting Information. The solutions present t^+^ values between 0.50 and 0.55 which suggest fast Li^+^ transport, although a decreasing trend is observed by the increase of DOL concentration in line with the increment of *E*_a_. Additional information about the salt dissolution in the electrolytes is provided by the corresponding FT-IR spectra in Figure 1g. The solutions show slight differences, whereas shifts are observed for the bands related to pure LiTFSI.^58^ In particular, the S–N–S stretching observed at 810, 773, and 774 cm^–1^ and the SO_2_ group asymmetric stretching at 1200 cm^–1^ move to lower wavenumbers, indicating the dissociation of the salt.^59,60^ Further proof of LiTFSI dissolution is given by the variation of the relative intensities with shift to higher wavenumbers of the peaks at 1350 and 1320 cm^–1^ accounting for CF_3_ asymmetric stretching.^58,60^ On the other hand, the increase in relative intensity of the signal near 1650 cm^–1^ attributed to LiTFSI by the DOL concentration raising may suggest a higher ion-pair association degree for TE-15% and TE-10% compared to TE-5%, in agreement with the respective lower values of ε_w_ discussed above.

**Table 2:** Parameters Used in Eq 2 to Evaluate the Li^+^ Transference Number (t^+^) through the Bruce–Vincent–Evans Method^48^^a^

electrolyte	*R*_0_ [Ω]	*R*_ss_ [Ω]	*i*_0_ [A]	*i*_ss_ [A]	*t*^+^
TE-5%	61.4	59.5	2.31 × 10^–^^4^	1.63 × 10^–^^4^	0.55
TE-10%	46.8	45.2	2.75 × 10^–^^4^	1.85 × 10^–^^4^	0.53
TE-15%	54.5	45.2	3.09 × 10^–^^4^	2.29 × 10^–^^4^	0.50

a Chronoamperometric curves and Nyquist plots used to determine values of current (*i*_0_ and *i*_ss_) and interphase resistance (*R*_0_ and *R*_ss_), respectively, are displayed in Figure S1 in the Supporting Information. See Experimental Section for details and Table 1 for electrolyte acronyms.

**Figure 2:**
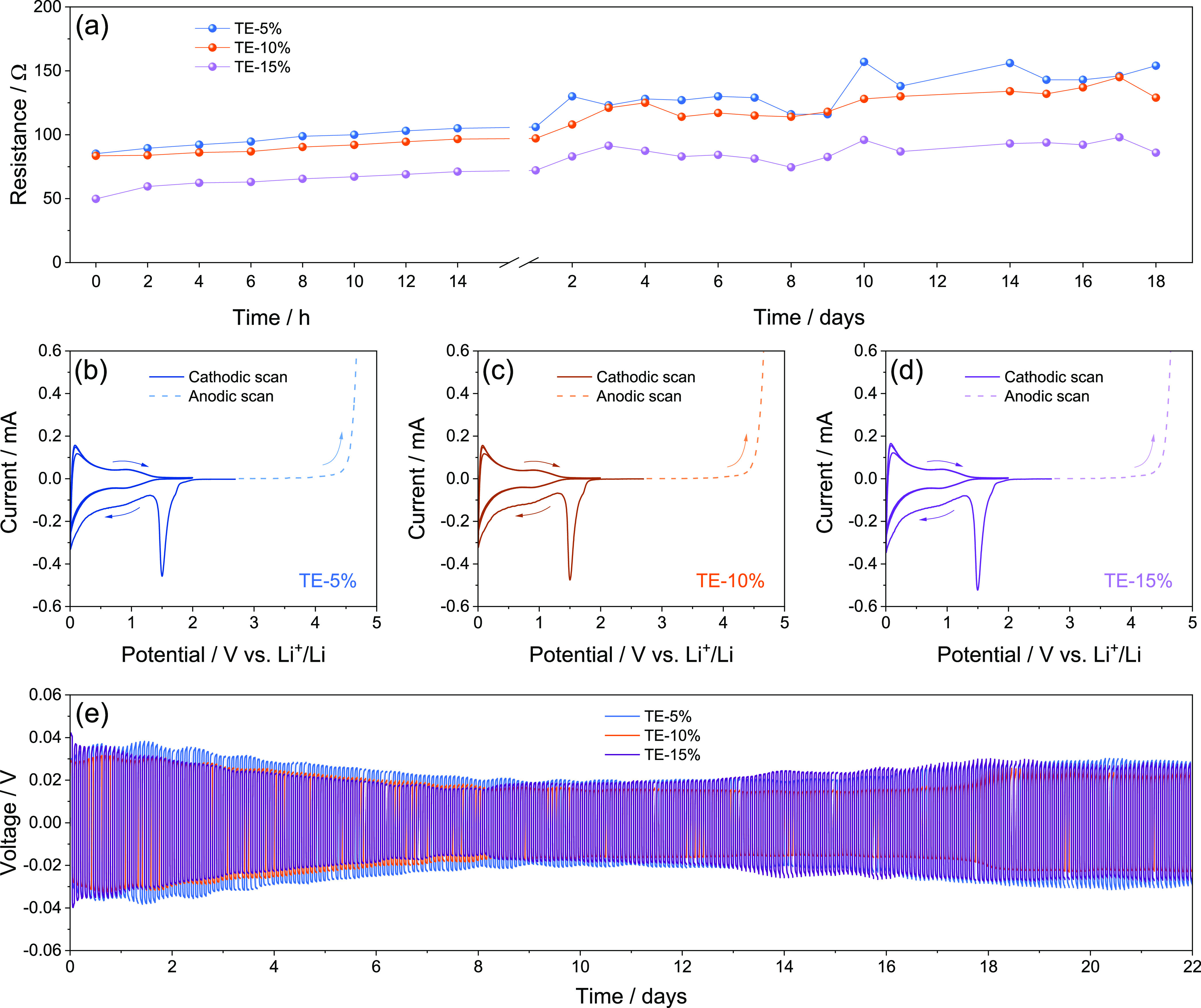
(a) Interphase resistance trends related to Li|Li cells using either TE-5%, TE-10%, or TE-15% aged for 18 days; see the corresponding Nyquist plots in Figure S2 and NLLS analyses in Tables S1–S3 in the Supporting Information; (b–d) ESW evaluation of the (b) TE-5%, (c) TE-10%, and (d) TE-15% electrolytes performed via CV in the cathodic region (0.01–2.0 V vs Li^+^/Li) and LSV in the anodic one (from OCV to 5.0 V vs Li^+^/Li) at a scan rate of 0.1 mV s^–1^; (e) lithium stripping-deposition tests performed on Li|Li cells using either TE-5%, TE-10%, or TE-15%. Figure S3 in the Supporting Information reports magnifications of the anodic stability curves and lithium stripping-deposition tests. See Table 1 for electrolyte acronyms.

The stability of the electrolytes in the Li cell is investigated in Figure 2 by monitoring the electrode/electrolyte interphase resistance upon cell aging (Figure 2a), evaluation of the ESW (Figure 2b–d), and Li stripping-deposition ability (Figure 2e). Figure 2a shows the electrode/electrolyte interphase resistance (*R*_i_) trends obtained by the NLLS analyses performed on the Nyquist plots reported in Figure S2 in the Supporting Information. The EIS spectra of the symmetrical Li|Li cells are plotted through the *R*_e_(*R*_i_*Q*_i_)(*R*_w_*Q*_w_) equivalent circuit, as displayed in Tables S1–S3 in the Supporting Information for TE-5%, TE-10%, and TE-15%, respectively (see Experimental Section for details).^49,50^ The results reveal initial *R*_i_ values of 85 Ω for TE-5%, 84 Ω for TE-10%, and 50 Ω for TE-15%, which raises upon cells aging to reach respective values of 154, 129, and 86 Ω after 18 days. The progressive increase and sporadic decreases of the interphase resistance are associated with growth and partial dissolution of the SEI on the lithium surface, leading to stabilization of the passivation layer and protection of the alkali metal.^61^ Interestingly, TE-5% exhibits generally higher values and lower stability of *R*_i_, followed by TE-10% and TE-15%, thus suggesting that the DOL can lead to a favorable SEI upon the statical storage.^53^Figure 2b–d displays the voltammograms recorded via CV between 0.01 and 2.0 V vs Li^+^/Li (cathodic scan) and LSV from OCV condition to 5.0 V vs Li^+^/Li (anodic scan) on Li|SPC cells to determine the ESW of TE-5% (Figure 2b), TE-10% (Figure 2c), and TE-15% (Figure 2d). The CV profiles of the cathodic scan show for all the solutions a sharp signal at 1.5 V vs Li^+^/Li during the first cycle ascribed to LiNO_3_ reduction,^62^ followed by a potential shoulder below 1.0 V vs Li^+^/Li and a final signal at 0.01 V vs Li^+^/Li accounting for partial electrolyte decomposition, Li insertion in the carbon matrix, and possible beginning of the Li electrodeposition.^55^ The subsequent cycles reveal low polarization of the reversible (de)insertion of Li in the carbon-based electrode, with notable stability suggested by the overlapping of the profiles. Therefore, the similar CV responses indicate only a marginal effect of DOL on the electrolyte cathodic stability. On the other hand, the LSV curves evidence that DOL addition leads to a different anodic stability, which is considered as the potential for which a non-negligible current of 30 μA is measured. Accordingly, Figure S3a (Supporting Information) provides a higher magnification of the anodic scans, revealing stability limits of 4.41 V vs Li^+^/Li for TE-5%, 4.38 V vs Li^+^/Li for TE-10%, and 4.37 V vs Li^+^/Li for TE-15%. The lower anodic stability triggered by DOL addition to TEGDME is actually expected due to the higher reactivity of the ether ring of the former compared to the glyme chain of the latter.^44^ Nevertheless, all the electrolytes provide ESWs extending from 0 to around 4.4 V vs Li^+^/Li, that is, a well sufficient span to host the Li–S electrochemical process.^19^ The lithium stripping-deposition profiles reported in Figure 2e are acquired from Li|Li cells to evaluate the overvoltage related to Li^+^ exchange through the electrolytes, which reflects the resistance of the interphase under dynamic conditions. During the initial cycles, all the solutions present a square-shape overvoltage with values approaching 40 mV (see magnification in Figure S3b in the Supporting Information), despite a slightly higher polarization being observed for TE-15% with respect to the other solutions. This may be attributed to the reactivity of the cyclic ether on the Li surface to form the SEI layer,^44^ which would lead to a more relevant overvoltage in TE-15% than in TE-5% and TE-10% at the beginning of the test due to the higher content of DOL in the solution. The polarization decreases after 7 days of test due to a partial SEI dissolution, and the overvoltage reaches values of 20, 23, and 27 mV for TE-15%, TE-10%, and TE-5%, respectively, at the beginning of the 8^th^ day (see magnification in Figure S3c in the Supporting Information). Despite the small differences, the more remarked polarization decrease for TE-15% with respect to TE-10% and TE-5% (see the comparison in Figure S3d in the Supporting Information) may account for a higher solubilization degree of the SEI possibly promoted by the lowered viscosity of the solvent mixture. On the other hand, the slight increase and stabilization of the overvoltage to values of ∼30 mV observed for all the solutions in the second half of testing are in line with the consolidation of a suitable SEI layer. The physical–chemical properties of TE-5%, TE-10%, and TE-15% are compared with those of DOL:DME and glyme-based solutions in Table S4 of the Supporting Information according to the previous literature.^13,18,19,53^ The data reveal the achievement of tuned properties between TEGDME and DOL:DME-based electrolytes, confirming the solutions studied herein as a possible step toward new electrolyte media combining the efficiency of DOL:DME and the safety of TEGDME.

**Figure 3:**
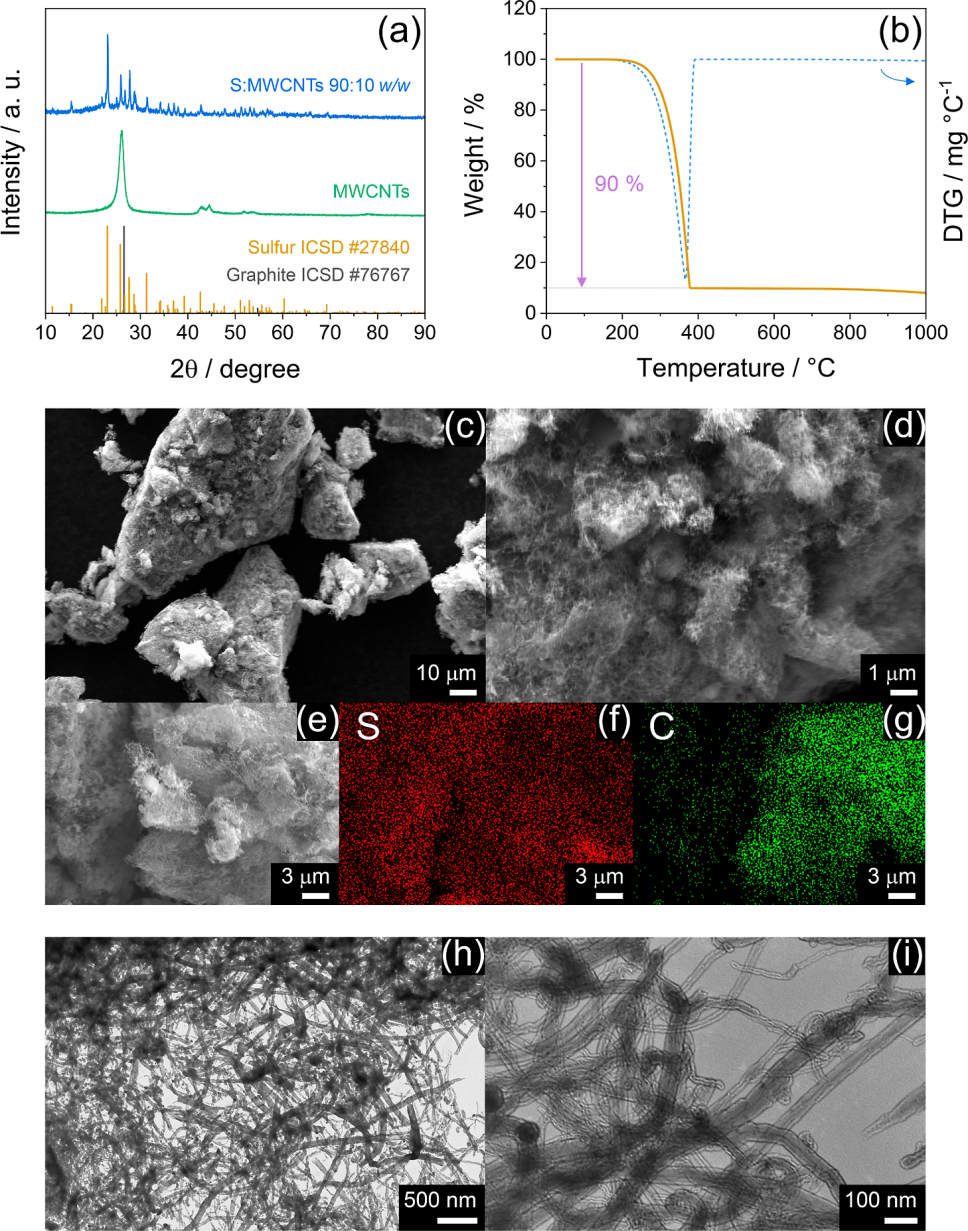
Physical–chemical characterization of the S:MWCNTs 90:10 w/w composite. In detail: (a) XRD of the composite and bare MWCNTs; reference data for sulfur (ICSD #27840) and graphite (ICSD #76767) are reported for comparison; (b) TGA and the corresponding DTG (right *y*-axis) performed under N_2_ flow between 25 and 1000 °C at the 5 °C min^–1^ rate; (c,d) SEM images at various magnification recorded in secondary electron mode; (e) SEM picture acquired in backscattered electrons mode and (f,g) corresponding elemental maps of (f) sulfur and (g) carbon; (h,i) TEM images at various magnifications.

The S:MWCNTs 90:10 w/w composite powder is investigated in Figure 3 in terms of structure, thermal behavior, and morphology by XRD (Figure 3a), TGA (Figure 3b), and SEM-EDS/TEM (Figure 3c–i), respectively. The X-ray diffractogram of the composite in Figure 3a shows the crystalline signature of orthorhombic sulfur according to the reference data (ICSD #27840 shown for comparison). The same figure reports the pattern of MWCNTs powder included in the electrode formulation and clearly reveals the graphitic character of this carbon (ICSD #76767 shown for comparison) with a main peak around 26°,^25^ which is instead observed as a broad wave extending from about 16–35° in the composite. The absence of additional signals accounting for undesired species excludes byproducts possibly formed during material synthesis which can lead to side reactivity during the Li–S conversion process, and confirms the suitability of the sulfur–carbon melt-mix process conducted at mild temperature. The success of the sulfur composite preparation is further confirmed by TGA and the corresponding DTG in Figure 3b, which reveals a sole weight loss between 200 and 380 °C accounting for the sulfur evaporation with a ratio of 90% of the total mass, exactly corresponding to the predicted amount.^63^ The SEM images acquired in secondary electron mode in Figure 3c,d show large sulfur clusters ranging from 10 to 100 μm (Figure 3c) formed by submicrometric primary particles covered by a thin layer of MWCNTs (Figure 3d). The uniform coverage of MWCNTs is highlighted by the SEM image recorded in backscattered electron mode in Figure 3e and by the corresponding EDS elemental mapping of sulfur and carbon in Figure 3f,g, respectively, which remark the efficient disposition of MWCNTs around the sulfur particles. Additional insight into the MWCNTs morphology and disposition in the S:MWCNTs 90:10 w/w composite is provided by the TEM images in Figure 3h,i. The micrographs evidence an entangled view of the system, which depicts a network of carbon across the sulfur clusters composed by connected nanotubes with micrometric length and thickness below 100 nm. Despite the relatively low ratio used herein, the conductive carbon net of the MWCNTs is expected to facilitate the electron pathway during electrochemical conversion, leading to satisfactory performance of the Li–S cell. On the other hand, the micrometric features of the composite can lower the impact of the side processes associated with the electrolyte decomposition on the overall reversibility of the main electrochemical reaction, and the high amount of the sulfur may ensure a high practical capacity and scalability of the composite material.^63^

**Figure 4:**
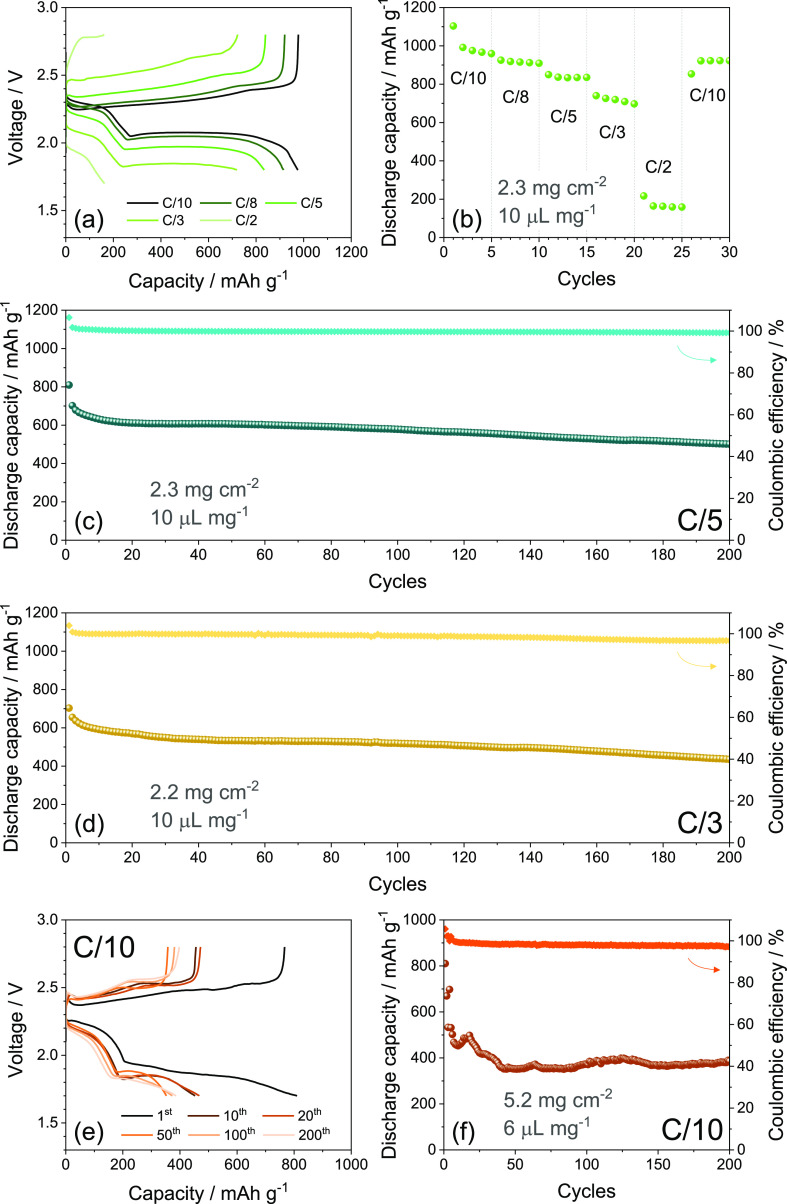
Galvanostatic cycling of Li cells using the S:MWCNTs 90:10 w/w electrode and the DOL:DME-control electrolyte with sulfur loading of 2.2–2.3 mg cm^–2^ and E/S ratio of 10 μL mg^–1^. In particular: (a) voltage profiles and the (b) corresponding capacity trend related to the rate capability test carried out at increasing scan rates from C/10 to C/2 between 1.8 and 2.8 V from C/10 to C/3 and in the 1.7–2.8 V voltage range for C/2; current rate was lowered back to C/10 after 25 cycles; (c,d) capacity trends (right *y*-axis reports Coulombic efficiency) recorded at constant current rates of either (c) C/5 or (d) C/3 between 1.7 and 2.8 V (see voltage profiles in Figure S4 of the Supporting Information); (e) voltage profiles and the (f) corresponding capacity trends (right *y*-axis reports Coulombic efficiency) acquired at C/10 between 1.7 and 2.8 V using sulfur loading increased to 5.2 mg cm^–2^ and E/S ratio limited to 6 μL mg^–1^.

The S:MWCNTs 90:10 w/w composite is subsequently included in a cathode and cycled in a lithium cell using the DOL:DME-control electrolyte, as reported in Figure 4. The voltage profiles of the rate capability test displayed in Figure 4a,b show the typical signature of the Li–S conversion process, where two galvanostatic discharge plateaus at 2.3 and 2.1 V are reflected during subsequent charge in two merging steps at 2.3 and 2.4 V.^64−66^ The discharge plateau at the higher voltage (i.e., 2.3 V) actually accounts for the initial conversion of Li and S to long chain polysulfides such as Li_2_S_8_, instead the one at the lower voltage (∼2.1 V) reflects the complex equilibrium including intermediate radical species during which the polysulfides are shortened to form Li_2_S_4_, Li_2_S_2_, and possibly Li_2_S by subsequent reductions.^64−66^ During the charge process, the oxidation back of the Li_2_S_*x*_ polysulfides occurs with a different pathway compared to the discharge process since the two steps discussed above are almost convoluted.^64−66^ As expected by the raising of C-rate from C/10 to C/3, the overvoltage between discharge and charge processes increases leading to a modest decrease of the delivered capacity; instead, the current of C/2 almost completely hinders the proper development of the low-voltage discharge plateau. Indeed, the corresponding discharge capacity trend (Figure 4b) evidences an initial value of 1100 mA h g^–1^ at C/10 and subsequent steady-state capacities of 976, 915, 833, 720, and 163 mA h g^–1^ at C/10, C/8, C/5, C/3, and C/2, respectively. Thus, a satisfactory rate capability is achieved by the S:MWCNTs 90:10 w/w from C/10 to C/3, as also suggested by the final capacity recovering at 922 mA h g^–1^ by lowering back the current at C/10 in the last five cycles, that is, 84% of the initial capacity and 94% compared to the steady state at the same C-rate. Instead, the very modest capacity of the cell at C/2 can be expected due to the relatively high amount of the active sulfur compared to electrochemically inactive elements such as the conductive carbon both in the composite and in the support, as well as by the laminated configuration of the electrode which can lead in turns to a relevant volumetric energy density.^25^ Prolonged galvanostatic cycling tests are carried out at the constant C/5 and C/3 rates, as reported in terms of capacity trends in Figure 4c,d, respectively, while the related voltage profiles are displayed in Figures S4 in the Supporting Information. The cell cycled at C/5 (Figure 4c) delivers an initial capacity of 810 mA h g^–1^ that decreases and stabilizes at about 610 mA h g^–1^ during the first 20 cycles likely due to consolidation of electrode/electrolyte interphase with SEI formation and partial loss of active material.^25^ On the other hand, the cell delivers 200 cycles with a final capacity of 500 mA h g^–1^, which corresponds to a retention of 62% of the initial value and 82% of the steady state and Coulombic efficiency exceeding 99% for the whole test. The test at C/3 (Figure 4d) shows a similar behavior, with an initial capacity of 703 mA h g^–1^, a steady state value of about 530 mA h g^–1^, and a final one of 436 mA h g^–1^ after 200 cycles, leading to the same retention observed for the measurement at C/5 and a Coulombic efficiency higher than 97%. These outcomes indicate the S:MWCNTs 90:10 w/w cathode as a viable solution to explore advanced configuration of Li–S batteries, as also suggested by the corresponding voltage profiles in Figure S4 of the Supporting Information which display full development of the reversible Li–S conversion process at both C/5 (Figure S4a) and C/3 (Figure S4b) rates with limited increase of polarization during cycling. The reversibility of the Li–S process is further demonstrated by the CV tests reported in Figure S5 in the Supporting Information. The voltammogram shows at the first cycle two reduction steps at 2.25 and 1.95 V vs Li^+^/Li reflected in a broad and convoluted double-charge step centered around 2.5 V vs Li^+^/Li in line with the reduction of Li and S to Li_2_S_*x*_ intermediates during discharge and their conversion back to Li and S during the subsequent charge process, respectively, as observed in galvanostatic cycling.^64−66^ The subsequent profiles show slight decrease of the signal intensity and shift of the reduction peaks due to a partial sulfur loss and consolidation of the electrode/electrolyte interphase upon initial stage, while the notable overlapping of the curves confirm the stability of the Li–S conversion process.^25^ Afterward, more challenging cycling conditions are adopted to evaluate the practical applicability of the S:MWCNTs 90:10 w/w electrode in Figure 4e,f, which displays a galvanostatic test performed with sulfur loading increased to 5.2 mg cm^–2^ and E/S ratio limited to 6 μL mg^–1^ using a current rate of C/10. The voltage profiles (Figure 4e) reveal higher polarization with respect to the previous tests and sloped discharge/charge plateaus, as expected by a higher resistance and lower wettability due to the incremented loading of the insulant active material and low E/S ratio that may partially hinder the liquid–solid conversion of the Li_2_S_*x*_ intermediate species. Accordingly, the decrease of capacity observed from 803 mA h g^–1^ at the first cycle to 531 mA h g^–1^ at the fifth is ascribed to the shortening of the low-voltage discharge plateau, associated with the conversion of the soluble long-chain polysulfides, such as Li_2_S_8_ and Li_2_S_6_, to the solid short-chain ones, such as Li_2_S_4_ and Li_2_S_2_.^64−66^ On the other hand, the cell delivers 200 cycles with maximum capacity approaching 500 mA h g^–1^ after the initial stage, which corresponds to a practical value of 4 mA h and an areal one of 2.6 mA h cm^–2^ referred to the geometric area of the electrode (1.54 cm^2^). Moreover, the cell exhibits a stable capacity between 350 and 400 mA h g^–1^ at the steady state maintained until the end of the test, as well as a Coulombic efficiency over 97%.

**Figure 5:**
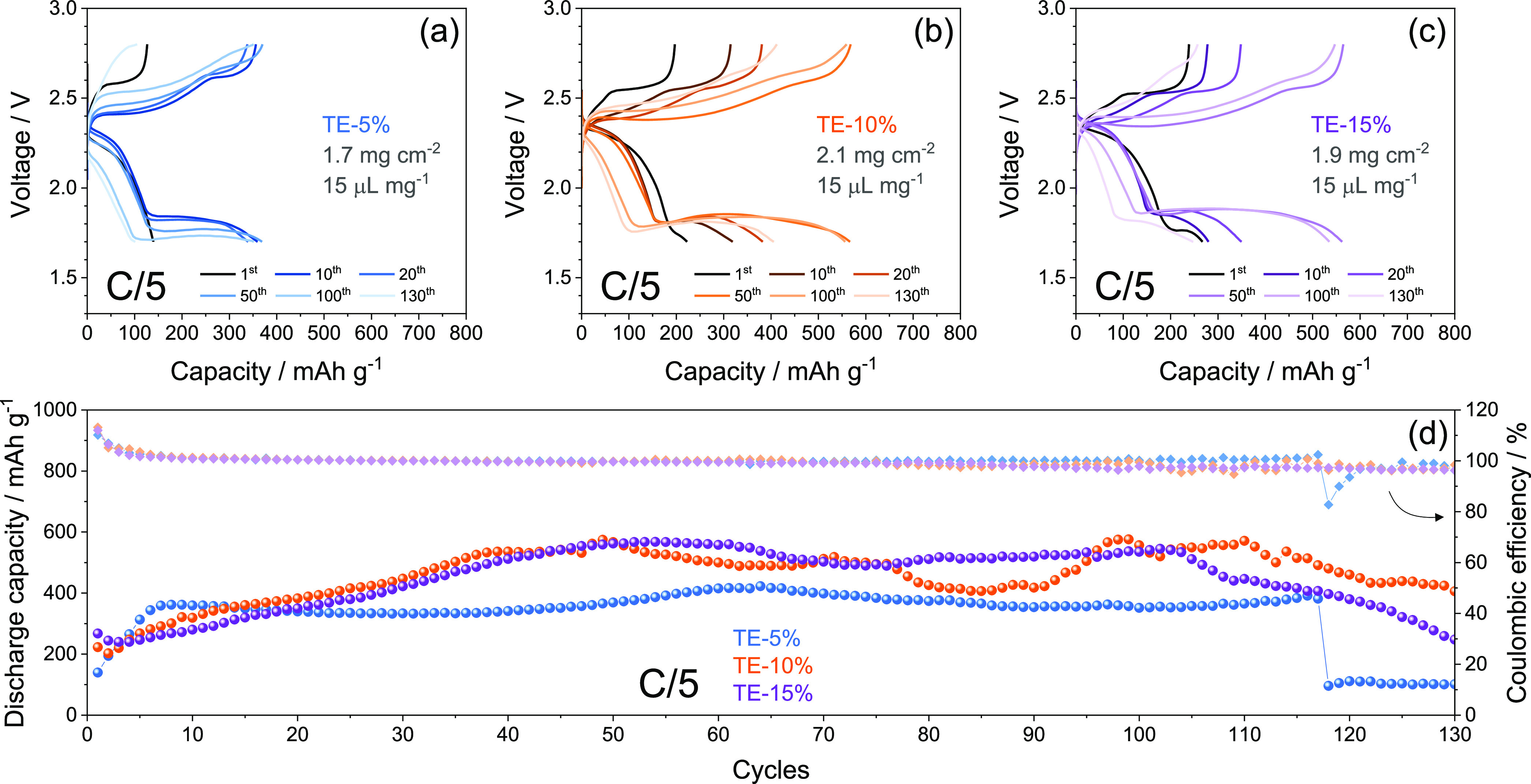
(a–c) Voltage profiles and (d) cycling trends (right *y*-axis shows Coulombic efficiency) of Li–S cells galvanostatically cycled at C/5 constant rate using either the (a) TE-5%, (b) TE-10%, or (c) TE-15% electrolyte and the S:MWCNTs 90:10 w/w electrode. Sulfur loading: 1.7–2.1 mg cm^–2^; E/S ratio: 15 μL mg^–1^; voltage range: 1.7–2.8 V.

Li–S cells using the S:MWCNTs 90:10 w/w electrode with TE-5%, TE-10%, and TE-15% electrolytes are cycled at the constant current rate of C/5 and reported in Figure 5. The voltage profiles in Figure 5a–c show that at the first cycle for all the cells, excessive polarization hinders the low-voltage discharge plateau. The above absence of the low-voltage plateau in TE-5% (Figure 5a), its partial development in TE-10% (Figure 5b), and its presence in TE-15% (Figure 5c) account for the enhancement of the liquid–solid conversion from soluble long-chain polysulfides to short-chain ones promoted by the decrease of viscosity of the electrolyte by DOL addition.^64−66^ On the other hand, all the cells present progressive activation of the Li–S conversion process indicated by the occurrence of the low-voltage discharge step upon cycling. Different activations depending on DOL concentration are evidenced by the cycling trends in Figure 5d. The figure displays an increase of the delivered cell capacity during the initial stage from 140 to 360 mA h g^–1^ for TE-5%, from 220 to 530 mA h g^–1^ for TE-10%, and from 270 to 570 mA h for TE-15%. Interestingly, the capacity growth speed decreases from TE-5% to TE-15%, thus suggesting a faster cell activation for lower DOL concentration. Despite the fact that decreasing viscosity from TE-5% to TE-15% can in principle allow a faster wetting of the sulfur electrode and facilitate the electrochemical process, the initial activation appears to be mostly controlled by *E*_a_ and ε_w_ values. In fact, Figure 5d evidences a capacity growth speed decreasing from TE-5% to TE-15%. This outcome may indicate that the activation speed is intimately correlated with the Li^+^ transport properties of the solution, which are relevantly influenced by *E*_a_ and ε_w_. Therefore, the progressively slower activation from TE-5% to TE-15% is reasonably explained by the respective decreases in *E*_a_ and ε_w_ (Figure 1). However, the low concentration of the cosolvent in TE-5% limits the discharge capacity of the cell to a maximum of 420 mA h g^–1^ and promotes the discharge/charge overvoltage, leading to a sudden deactivation of the electrochemical process after 117 cycles with capacity below 100 mA h g^–1^ (Figure 5d). On the other hand, TE-15% allows in the cell a stable, steady-state capacity between 500 and 550 mA h g^–1^, although a gradual deactivation occurs after 100 cycles to a final capacity of 250 mA h g^–1^ (Figure 5d). The cell using TE-10% reveals a fluctuation of the capacity between 400 and 580 mA h g^–1^ and a decrease limited to 406 mA h g^–1^ after 130 cycles (Figure 5d). Interestingly, all the cells have a Coulombic efficiency exceeding 96% during the whole test, except the one with TE-5% showing efficiency decrease to 82% in correspondence to conversion deactivation. The fluctuations of the discharge capacity observed in Figure 5d may be due to complex interplay during cycling between TEGDME, DOL, the formed cosolvents, and the Li_2_S_*x*_ intermediates, depending on the TEGDME:DOL ratio. Hence, TE-10% presents the most relevant fluctuations with the most improved cycle life, thus suggesting that the addition of 10 wt % DOL particularly acts on the polysulfides solvation and nature. On the other hand, TE-5% and TE-15% appear to mainly affect the reaction kinetics and the film formation, in particular during the final stages of the cycling tests. Indeed, TE-5% exhibits stable discharge capacity in line with the beneficial effects of the glyme solvent which guarantees an efficient Li^+^ exchange, but the excessive viscosity of the solution limits both capacity value and cycle life. Furthermore, TE-15% delivers higher capacity, thanks to the limited viscosity, but the continuous depletion of DOL likely causes irregular and excessive SEI that shortens the cycle life. Hence, the data of Figure 5 evidence a complex interplay between DOL and TEGDME in the Li–S cell. The relevant amount of DOL in TE-15% mitigates the solution viscosity but slows the speed of the Li–S activation. At the same time, the DOL leads in the TE-15% cell to the higher steady-state capacity, however, with excessive SEI growth affecting the cycle life as evidenced by the capacity deactivation after 110 cycles. In spite, the low DOL content in TE-5% ensures a fast activation to the cell likely due to the enhanced Li^+^ exchange allowed by the lower *E*_a_ and ε_w_ (see Figure 1) but limits the discharge capacity and leads to cell failure after 117 cycles due to the excessive polarization raise attributed to the relevant viscosity of the solution. The TE-10% appears to be the most promising compromise since the corresponding Li–S cell shows the longer cycle life likely ascribed to the optimal proportion between TEGDME and DOL, despite the fluctuation of the capacity during cycling, which may be assessed by improving the cathode configuration. Table S5 in the Supporting Information reports a comparison between the performance achieved by TE-10% and literature data on common DOL:DME electrolytes and glyme-based solutions.^12,14,16,25^ The table shows that our results are in line with previous Li–S reports and include the additional bonuses of enhanced sulfur loading in the composite and relevant safety of the cell. The rate capability of the Li–S cells is further evaluated in the Figure S6 of the Supporting Information. The voltage profiles (Figure S6a–c) and the corresponding cycling trend (Figure S6d) reveal full development of the Li–S conversion process at C/20, C/10, and C/8 for all the solutions with steady-state capacities of 920, 810, and 725 mA h g^–1^ for TE-15%, 880, 735, and 610 mA h g^–1^ for TE-10%, and 900, 760, and 670 mA h g^–1^ for TE-5%, respectively. On the other hand, only TE-15% and TE-10% exhibit an acceptable response at C/5 showing capacities of 640 and around 500 mA h g^–1^, respectively, while the capacity delivered by TE-5% is limited to 150 mA h g^–1^. In addition, all the electrolytes show poor performance at C/3 with a maximum capacity of 130 mA h g^–1^ related to TE-15%, while deactivation of the electrochemical process is observed at C/2. The rate capability tests demonstrate the improvement of conversion kinetics by DOL increase; however, the same tests reveal a poor rate capability and the need for further focus on the solution design in order to achieve satisfactory performance at high currents which is heavily influenced by the viscosity of the electrolyte. Moreover, the outcomes shed light on the necessity of a proper activation by prolonged cycling due to the poor capacity delivered by TE-5% at C/5 and to the relevant sloping trend of TE-10%. In summary, the tuning of the amount of DOL cosolvent in the viscous glyme-based electrolytes investigated herein may pave the way toward optimized compositions where low flammability, negligible toxicity, and performance are thoroughly balanced to achieve safe and scalable Li–S batteries. Certainly, further dedicated studies are necessary in order to achieve the optimal combinations of cosolvents and ad hoc concentrations of additives.

## 4 Conclusions

The TE-5%, TE-10%, and TE-15% electrolytes revealed the absence of ignition upon direct exposure to flame, thus suggesting a remarkable safety content. The electrolytes exhibited stability up to 200 °C under N_2_ and a thermal trend influenced by DOL. The suitable ionic conductivity of the electrolytes from −3 to 50 °C allowed the estimation of the activation energy for Li^+^ motion, depending on the DOL content. The Li^+^ transference number of the solutions decreased from 0.55 to 0.50 from TE-5% to TE-15% due to the lower dielectric constant in the latter compared to the former, which also influenced the LiTFSI dissolution. On the other hand, the increment of DOL concentration lowered the electrode/electrolyte interphase resistance in the Li cell that reached the lowest values for TE-15% upon aging. All solutions revealed a cathodic limit of 0 V vs Li^+^/Li and anodic limit decreasing from 4.41 V for TE-5%, to 4.38 V for TE-10%, and to 4.37 V for TE-15% due to the reactivity of the DOL ring. Li stripping/deposition tests have shown modest charge/discharge overvoltage and the lowest polarization for TE-15%. A composite cathode including 90 wt % sulfur with a micrometric shape and a submicron primary arrangement of MWCNT network revealed in a DOL:DME control-electrolyte a maximum capacity of ∼1100 mA h g^–1^, with life extended up to 200 cycles and Coulombic efficiency ∼100%. Similar control cells with sulfur loading increased to 5.2 mg cm^–2^ and E/S ratio decreased to 6 μL mg^–1^ exhibited a maximum capacity of 500 mA h g^–1^ (4 mA h and 2.6 mA h cm^–2^), life of 200 cycles, and Coulombic efficiency above 97%. Subsequently, the above electrode and the investigated glyme-based electrolytes are combined in a safe Li–S cell. The lowest DOL concentration (TE-5%) has allowed a fast cell activation, however, with capacity limited to 420 mA h g^–1^ and a sudden deactivation due to a polarization increase. Instead, the highest DOL content (TE-15%) promoted a capacity exceeding 550 mA h g^–1^, however, with a gradual decrease after 100 cycles due to the excessive SEI growth. Likely, TE-10% represented an optimal compromise, showing fluctuations of the delivered capacity between 400 and 580 mA h g^–1^ and a life of 130 cycles.

## Supporting Information Available

EIS Nyquist plots to calculate the ionic conductivity and the chronoamperometric curves with Nyquist plots to calculate the Li^+^ transference number of the electrolytes; EIS Nyquist plots and the corresponding NLLS analyses for the evaluation of the Li-electrolyte resistance; comparison of the physical–chemical properties of solutions with other electrolytes employed in the Li–S cell in the previous literature; galvanostatic voltage profiles and CV curves of Li–S cells using the S:MWCNTs 90:10 w/w electrode and the DOL:DME-control electrolyte; comparison of the electrochemical performance obtained from the Li–S cell studied in this work with previous literature papers; rate capability tests of Li–S cells using the electrolytes (PDF)
Movie S1: Flammability tests of the electrolytes TE-5% (MP4)
Movie S2: Flammability tests of the electrolytes TE-10% (MP4)
Movie S3: Flammability tests of the electrolytes TE-15% (MP4)
Movie S4: Flammability tests of DOL:DME-control electrolyte (MP4)

## References

[ref1] Schipper F.; Erickson E. M.; Erk C.; Shin J.-Y.; Chesneau F. F.; Aurbach D. Review—Recent Advances and Remaining Challenges for Lithium Ion Battery Cathodes. J. Electrochem. Soc. 2017, 164 (1), A6220–A6228. 10.1149/2.0351701jes.

[ref2] Choi J. W.; Aurbach D. Promise and Reality of Post-Lithium-Ion Batteries with High Energy Densities. Nat. Rev. Mater. 2016, 1, 16013 10.1038/natrevmats.2016.13.

[ref3] Fotouhi A.; Auger D. J.; Propp K.; Longo S.; Wild M. A Review on Electric Vehicle Battery Modelling: From Lithium-Ion toward Lithium-Sulphur. Renewable Sustainable Energy Rev. 2016, 56, 1008–1021. 10.1016/j.rser.2015.12.009.

[ref4] Scrosati B.; Hassoun J.; Sun Y.-K. Lithium-Ion Batteries. A Look into the Future. Energy Environ. Sci. 2011, 4 (9), 3287–3295. 10.1039/c1ee01388b.

[ref5] Wang W.; Wang Y.; Huang Y.; Huang C.; Yu Z.; Zhang H.; Wang A.; Yuan K. The Electrochemical Performance of Lithium-Sulfur Batteries with LiClO_4_ DOL/DME Electrolyte. J. Appl. Electrochem. 2010, 40 (2), 321–325. 10.1007/s10800-009-9978-z.

[ref6] Carbone L.; Greenbaum S. G.; Hassoun J. Lithium Sulfur and Lithium Oxygen Batteries: New Frontiers of Sustainable Energy Storage. Sustainable Energy Fuels 2017, 1 (2), 228–247. 10.1039/C6SE00124F.

[ref7] Lisbona D.; Snee T. A Review of Hazards Associated with Primary Lithium and Lithium-Ion Batteries. Process Saf. Environ. Prot. 2011, 89 (6), 434–442. 10.1016/j.psep.2011.06.022.

[ref8] Liu G.; Sun Q.; Li Q.; Zhang J.; Ming J. Electrolyte Issues in Lithium-Sulfur Batteries: Development, Prospect, and Challenges. Energy Fuels 2021, 35 (13), 10405–10427. 10.1021/acs.energyfuels.1c00990.

[ref9] Di Lecce D.; Marangon V.; Jung H.-G.; Tominaga Y.; Greenbaum S.; Hassoun J. Glyme-Based Electrolytes: Suitable Solutions for next-Generation Lithium Batteries. Green Chem. 2022, 24 (3), 1021–1048. 10.1039/D1GC03996B.

[ref10] Di Lecce D.; Marangon V.; Benítez A.; Caballero A. ´.; Morales J.; Rodríguez-Castellón E.; Hassoun J. High Capacity Semi-Liquid Lithium Sulfur Cells with Enhanced Reversibility for Application in New-Generation Energy Storage Systems. J. Power Sources 2019, 412, 575–585. 10.1016/j.jpowsour.2018.11.068.

[ref11] Adams B. D.; Carino E. V.; Connell J. G.; Han K. S.; Cao R.; Chen J.; Zheng J.; Li Q.; Mueller K. T.; Henderson W. A.; Zhang J.-G. Long Term Stability of Li-S Batteries Using High Concentration Lithium Nitrate Electrolytes. Nano Energy 2017, 40, 607–617. 10.1016/j.nanoen.2017.09.015.

[ref12] Pang Q.; Shyamsunder A.; Narayanan B.; Kwok C. Y.; Curtiss L. A.; Nazar L. F. Tuning the Electrolyte Network Structure to Invoke Quasi-Solid State Sulfur Conversion and Suppress Lithium Dendrite Formation in Li-S Batteries. Nat. Energy 2018, 3 (9), 783–791. 10.1038/s41560-018-0214-0.

[ref13] Carbone L.; Coneglian T.; Gobet M.; Munoz S.; Devany M.; Greenbaum S.; Hassoun J. A Simple Approach for Making a Viable, Safe, and High-Performances Lithium-Sulfur Battery. J. Power Sources 2018, 377 (September 2017), 26–35. 10.1016/j.jpowsour.2017.11.079.

[ref14] Seki S.; Serizawa N.; Takei K.; Umebayashi Y.; Tsuzuki S.; Watanabe M. Long-Cycle-Life Lithium-Sulfur Batteries with Lithium Solvate Ionic Liquids. Electrochemistry 2017, 85 (10), 680–682. 10.5796/electrochemistry.85.680.

[ref15] Ishino Y.; Takahashi K.; Murata W.; Umebayashi Y.; Tsuzuki S.; Watanabe M.; Kamaya M.; Seki S. Effect of Electrolyte Composition on Performance and Stability of Lithium-Sulfur Batteries. Energy Technol. 2019, 7 (12), 1900197 10.1002/ente.201900197.

[ref16] Lu H.; Yuan Y.; Hou Z.; Lai Y.; Zhang K.; Liu Y. Solvate Ionic Liquid Electrolyte with 1,1,2,2-Tetrafluoroethyl 2,2,2-Trifluoroethyl Ether as a Support Solvent for Advanced Lithium-Sulfur Batteries. RSC Adv. 2016, 6 (22), 18186–18190. 10.1039/C5RA24182K.

[ref17] Agostini M.; Xiong S.; Matic A.; Hassoun J. Polysulfide-Containing Glyme-Based Electrolytes for Lithium Sulfur Battery. Chem. Mater. 2015, 27 (13), 4604–4611. 10.1021/acs.chemmater.5b00896.

[ref18] Marangon V.; Di Lecce D.; Minnetti L.; Hassoun J. Novel Lithium Sulfur Polymer Battery Operating at Moderate Temperature. ChemElectroChem 2021, 8 (20), 3971–3981. 10.1002/celc.202101272.

[ref19] Marangon V.; Minnetti L.; Barcaro E.; Hassoun J. Room Temperature Solid State Polymer Electrolyte in Li LiFePO_4_, Li S and Li O_2_ Batteries. Chem.—Eur. J. 2023, 29, e202301345 10.1002/chem.202301345. 37203374

[ref20] Di Lecce D.; Carbone L.; Gancitano V.; Hassoun J. Rechargeable Lithium Battery Using Non-Flammable Electrolyte Based on Tetraethylene Glycol Dimethyl Ether and Olivine Cathodes. J. Power Sources 2016, 334, 146–153. 10.1016/j.jpowsour.2016.09.164.

[ref21] Bhargav A.; He J.; Gupta A.; Manthiram A. Lithium-Sulfur Batteries: Attaining the Critical Metrics. Joule 2020, 4 (2), 285–291. 10.1016/j.joule.2020.01.001.

[ref22] Cheng Q.; Chen Z.-X.; Li X.-Y.; Hou L.-P.; Bi C.-X.; Zhang X.-Q.; Huang J.-Q.; Li B.-Q. Constructing a 700 Wh kg-1-Level Rechargeable Lithium-Sulfur Pouch Cell. J. Energy Chem. 2023, 76, 181–186. 10.1016/j.jechem.2022.09.029.

[ref23] Pang Q.; Liang X.; Kwok C. Y.; Kulisch J.; Nazar L. F. A Comprehensive Approach toward Stable Lithium-Sulfur Batteries with High Volumetric Energy Density. Adv. Energy Mater. 2017, 7 (6), 1601630 10.1002/aenm.201601630.

[ref24] Shen Z.; Zhang W.; Mao S.; Li S.; Wang X.; Lu Y. Tailored Electrolytes Enabling Practical Lithium-Sulfur Full Batteries via Interfacial Protection. ACS Energy Lett. 2021, 6 (8), 2673–2681. 10.1021/acsenergylett.1c01091.

[ref25] Marangon V.; Barcaro E.; Minnetti L.; Brehm W.; Bonaccorso F.; Pellegrini V.; Hassoun J. Current Collectors Based on Multiwalled Carbon-Nanotubes and Few-Layer Graphene for Enhancing the Conversion Process in Scalable Lithium-Sulfur Battery. Nano Res. 2023, 16 (6), 8433–8447. 10.1007/s12274-022-5364-5.

[ref26] Brehm W.; Marangon V.; Panda J.; Thorat S. B.; del Rio Castillo A. E.; Bonaccorso F.; Pellegrini V.; Hassoun J. A Lithium-Sulfur Battery Using Binder-Free Graphene-Coated Aluminum Current Collector. Energy Fuels 2022, 36 (16), 9321–9328. 10.1021/acs.energyfuels.2c02086. 36016761 PMC9394755

[ref27] Li W.; Yao H.; Yan K.; Zheng G.; Liang Z.; Chiang Y.-M.; Cui Y. The Synergetic Effect of Lithium Polysulfide and Lithium Nitrate to Prevent Lithium Dendrite Growth. Nat. Commun. 2015, 6 (1), 7436 10.1038/ncomms8436. 26081242

[ref28] Arbizzani C.; Gabrielli G.; Mastragostino M. Thermal Stability and Flammability of Electrolytes for Lithium-Ion Batteries. J. Power Sources 2011, 196 (10), 4801–4805. 10.1016/j.jpowsour.2011.01.068.

[ref29] Guo F.; Hase W.; Ozaki Y.; Konno Y.; Inatsuki M.; Nishimura K.; Hashimoto N.; Fujita O. Experimental Study on Flammability Limits of Electrolyte Solvents in Lithium-Ion Batteries Using a Wick Combustion Method. Exp. Therm. Fluid Sci. 2019, 109, 109858 10.1016/j.expthermflusci.2019.109858.

[ref30] Balakrishnan P. G.; Ramesh R.; Prem Kumar T. Safety Mechanisms in Lithium-Ion Batteries. J. Power Sources 2006, 155 (2), 401–414. 10.1016/j.jpowsour.2005.12.002.

[ref31] Wu Y.; Wang W.; Ming J.; Li M.; Xie L.; He X.; Wang J.; Liang S.; Wu Y. An Exploration of New Energy Storage System: High Energy Density, High Safety, and Fast Charging Lithium Ion Battery. Adv. Funct. Mater. 2019, 29 (1), 1805978 10.1002/adfm.201805978.

[ref32] Marangon V.; Di Lecce D.; Brett D. J. L.; Shearing P. R.; Hassoun J. Characteristics of a Gold-Doped Electrode for Application in High-Performance Lithium-Sulfur Battery. J. Energy Chem. 2022, 64, 116–128. 10.1016/j.jechem.2021.04.025.

[ref33] Marangon V.; Scaduti E.; Vinci V. F.; Hassoun J. Scalable Composites Benefiting from Transition Metal Oxides as Cathode Materials for Efficient Lithium Sulfur Batteries. ChemElectroChem 2022, 9 (11), e202200374 10.1002/celc.202200374.

[ref34] Feng J.; Zheng D.; Yin R.; Niu X.; Xu X.; Meng S.; Ma S.; Shi W.; Wu F.; Liu W.; Cao X. A Wide Temperature Adaptive Aqueous Zinc Air Battery Based on Cu-Co Dual Metal-Nitrogen Carbon/Nanoparticle Electrocatalysts. Small Struct. 2023, 4, 2200340 10.1002/sstr.202200340.

[ref35] Liu W.; Niu X.; Feng J.; Yin R.; Ma S.; Que W.; Dai J.; Tang J.; Wu F.; Shi W.; Liu X.; Cao X. Tunable Heterogeneous FeCo Alloy-Mo_0.82_N Bifunctional Electrocatalysts for Temperature-Adapted Zn-Air Batteries. ACS Appl. Mater. Interfaces 2023, 15 (12), 15344–15352. 10.1021/acsami.2c21616. 36920344

[ref36] Liu W.; Que W.; Yin R.; Dai J.; Zheng D.; Feng J.; Xu X.; Wu F.; Shi W.; Liu X.; Cao X. Ferrum-Molybdenum Dual Incorporated Cobalt Oxides as Efficient Bifunctional Anti-Corrosion Electrocatalyst for Seawater Splitting. Appl. Catal., B 2023, 328, 122488 10.1016/j.apcatb.2023.122488.

[ref37] Wang W.; Cao Z.; Elia G. A.; Wu Y.; Wahyudi W.; Abou-Hamad E.; Emwas A.-H.; Cavallo L.; Li L.-J.; Ming J. Recognizing the Mechanism of Sulfurized Polyacrylonitrile Cathode Materials for Li-S Batteries and beyond in Al-S Batteries. ACS Energy Lett. 2018, 3 (12), 2899–2907. 10.1021/acsenergylett.8b01945.

[ref38] Ming J.; Li M.; Kumar P.; Li L.-J. Multilayer Approach for Advanced Hybrid Lithium Battery. ACS Nano 2016, 10 (6), 6037–6044. 10.1021/acsnano.6b01626. 27268064

[ref39] Ming J.; Li M.; Kumar P.; Lu A.-Y.; Wahyudi W.; Li L.-J. Redox Species-Based Electrolytes for Advanced Rechargeable Lithium Ion Batteries. ACS Energy Lett. 2016, 1 (3), 529–534. 10.1021/acsenergylett.6b00274.

[ref40] Tang S.; Zhao H. Glymes as Versatile Solvents for Chemical Reactions and Processes: From the Laboratory to Industry. RSC Adv. 2014, 4 (22), 11251 10.1039/c3ra47191h. 24729866 PMC3981120

[ref41] Ruiz Holgado M. E. F. de; Schaefer C. R. de; Arancibia E. L. Densities and Viscosities of Binary Mixtures of Polyethylene Glycol 350 Monomethyl Ether with n -Butanol and n -Pentanol and Tetraethylene Glycol Dimethyl Ethers with n -Propanol, n -Butanol, and n -Pentanol from 278.15 to 318.15 K. J. Chem. Eng. Data 2002, 47 (2), 144–148. 10.1021/je010182s.

[ref42] Park C.; Kanduč M.; Chudoba R.; Ronneburg A.; Risse S.; Ballauff M.; Dzubiella J. Molecular Simulations of Electrolyte Structure and Dynamics in Lithium-Sulfur Battery Solvents. J. Power Sources 2018, 373, 70–78. 10.1016/j.jpowsour.2017.10.081.

[ref43] Barchasz C.; Lepretre J.-C.; Patoux S.; Alloin F. Revisiting TEGDME/DIOX Binary Electrolytes for Lithium/Sulfur Batteries: Importance of Solvation Ability and Additives. J. Electrochem. Soc. 2013, 160 (3), A430–A436. 10.1149/2.022303jes.

[ref44] Aurbach D.; Pollak E.; Elazari R.; Salitra G.; Kelley C. S. S.; Affinito J. On the Surface Chemical Aspects of Very High Energy Density, Rechargeable Li-Sulfur Batteries. J. Electrochem. Soc. 2009, 156 (8), A694 10.1149/1.3148721.

[ref45] Kebede A. A.; Coosemans T.; Messagie M.; Jemal T.; Behabtu H. A.; Van Mierlo J.; Berecibar M. Techno-Economic Analysis of Lithium-Ion and Lead-Acid Batteries in Stationary Energy Storage Application. J. Energy Storage 2021, 40, 102748 10.1016/j.est.2021.102748.

[ref46] Levchenko S.; Wei S.; Marangon V.; Hassoun J. A Li-Ion Battery Using Nanostructured Sn@C Alloying Anode and High Voltage LiNi_0.35_Cu_0.1_Mn_1.45_Al_0.1_O_4_ Spinel Cathode. Energy Technol. 2022, 10 (12), 2200725 10.1002/ente.202200725.

[ref47] Laidler K. J. The Development of the Arrhenius Equation. J. Chem. Educ. 1984, 61 (6), 494 10.1021/ed061p494.

[ref48] Evans J.; Vincent C. A.; Bruce P. G. Electrochemical Measurement of Transference Numbers in Polymer Electrolytes. Polymer 1987, 28 (13), 2324–2328. 10.1016/0032-3861(87)90394-6.

[ref49] Boukamp B. A Package for Impedance/Admittance Data Analysis. Solid State Ionics 1986, 18–19, 136–140. 10.1016/0167-2738(86)90100-1.

[ref50] Boukamp B. A Nonlinear Least Squares Fit Procedure for Analysis of Immittance Data of Electrochemical Systems. Solid State Ionics 1986, 20 (1), 31–44. 10.1016/0167-2738(86)90031-7.

[ref51] Levi M. D.; Wang C.; Aurbach D. Two Parallel Diffusion Paths Model for Interpretation of PITT and EIS Responses from Non-Uniform Intercalation Electrodes. J. Electroanal. Chem. 2004, 561, 1–11. 10.1016/j.jelechem.2003.07.014.

[ref52] Del Rio Castillo A. E.; Pellegrini V.; Ansaldo A.; Ricciardella F.; Sun H.; Marasco L.; Buha J.; Dang Z.; Gagliani L.; Lago E.; Curreli N.; Gentiluomo S.; Palazon F.; Prato M.; Oropesa-Nuñez R.; Toth P. S.; Mantero E.; Crugliano M.; Gamucci A.; Tomadin A.; Polini M.; Bonaccorso F. High-Yield Production of 2D Crystals by Wet-Jet Milling. Mater. Horiz. 2018, 5 (5), 890–904. 10.1039/C8MH00487K.

[ref53] Carbone L.; Gobet M.; Peng J.; Devany M.; Scrosati B.; Greenbaum S.; Hassoun J. Comparative Study of Ether-Based Electrolytes for Application in Lithium-Sulfur Battery. ACS Appl. Mater. Interfaces 2015, 7 (25), 13859–13865. 10.1021/acsami.5b02160. 26057152

[ref54] Shimizu K.; Freitas A. A.; Atkin R.; Warr G. G.; FitzGerald P. A.; Doi H.; Saito S.; Ueno K.; Umebayashi Y.; Watanabe M.; Canongia Lopes J. N. Structural and Aggregate Analyses of (Li Salt + Glyme) Mixtures: The Complex Nature of Solvate Ionic Liquids. Phys. Chem. Chem. Phys. 2015, 17 (34), 22321–22335. 10.1039/C5CP03414K. 26245295

[ref55] Marangon V.; Hernandez-Rentero C.; Levchenko S.; Bianchini G.; Spagnolo D.; Caballero A.; Morales J.; Hassoun J. Lithium-Oxygen Battery Exploiting Highly Concentrated Glyme-Based Electrolytes. ACS Appl. Energy Mater. 2020, 3 (12), 12263–12275. 10.1021/acsaem.0c02331.

[ref56] Xu K. Nonaqueous Liquid Electrolytes for Lithium-Based Rechargeable Batteries. Chem. Rev. 2004, 104 (10), 4303–4418. 10.1021/cr030203g. 15669157

[ref57] Riadigos C. F.; Iglesias R.; Rivas M. A.; Iglesias T. P. Permittivity and Density of the Systems (Monoglyme, Diglyme, Triglyme, or Tetraglyme+n-Heptane) at Several Temperatures. J. Chem. Thermodyn. 2011, 43 (3), 275–283. 10.1016/j.jct.2010.09.008.

[ref58] Wong D. H. C.; Vitale A.; Devaux D.; Taylor A.; Pandya A. A.; Hallinan D. T.; Thelen J. L.; Mecham S. J.; Lux S. F.; Lapides A. M.; Resnick P. R.; Meyer T. J.; Kostecki R. M.; Balsara N. P.; DeSimone J. M. Phase Behavior and Electrochemical Characterization of Blends of Perfluoropolyether, Poly(Ethylene Glycol), and a Lithium Salt. Chem. Mater. 2015, 27 (2), 597–603. 10.1021/cm504228a.

[ref59] Rey I.; Lassègues J.; Grondin J.; Servant L. Infrared and Raman Study of the PEO-LiTFSI Polymer Electrolyte. Electrochim. Acta 1998, 43 (10–11), 1505–1510. 10.1016/S0013-4686(97)10092-5.

[ref60] Barteau K. P.; Wolffs M.; Lynd N. A.; Fredrickson G. H.; Kramer E. J.; Hawker C. J. Allyl Glycidyl Ether-Based Polymer Electrolytes for Room Temperature Lithium Batteries. Macromolecules 2013, 46 (22), 8988–8994. 10.1021/ma401267w.

[ref61] Aurbach D. Review of Selected Electrode-Solution Interactions Which Determine the Performance of Li and Li Ion Batteries. J. Power Sources 2000, 89 (2), 206–218. 10.1016/S0378-7753(00)00431-6.

[ref62] Zhang S. S. Role of LiNO_3_ in Rechargeable Lithium/Sulfur Battery. Electrochim. Acta 2012, 70, 344–348. 10.1016/j.electacta.2012.03.081.

[ref63] Páez Jerez A. L.; Chemes D. M.; Sham E. L.; Davies L. E.; Tesio A. Y.; Flexer V. Low Temperature Synthesis of a Sulfur Polyacrylonitrile Composite Cathode for Lithium Sulfur Batteries. ChemistrySelect 2020, 5 (18), 5465–5472. 10.1002/slct.202001529.

[ref64] Li G.; Wang S.; Zhang Y.; Li M.; Chen Z.; Lu J. Revisiting the Role of Polysulfides in Lithium-Sulfur Batteries. Adv. Mater. 2018, 30 (22), 1705590 10.1002/adma.201705590. 29577456

[ref65] Xiao J.; Hu J. Z.; Chen H.; Vijayakumar M.; Zheng J.; Pan H.; Walter E. D.; Hu M.; Deng X.; Feng J.; Liaw B. Y.; Gu M.; Deng Z. D.; Lu D.; Xu S.; Wang C.; Liu J. Following the Transient Reactions in Lithium-Sulfur Batteries Using an in Situ Nuclear Magnetic Resonance Technique. Nano Lett. 2015, 15 (5), 3309–3316. 10.1021/acs.nanolett.5b00521. 25785550

[ref66] Wang Q.; Zheng J.; Walter E.; Pan H.; Lv D.; Zuo P.; Chen H.; Deng Z. D.; Liaw B. Y.; Yu X.; Yang X.; Zhang J.-G.; Liu J.; Xiao J. Direct Observation of Sulfur Radicals as Reaction Media in Lithium Sulfur Batteries. J. Electrochem. Soc. 2015, 162 (3), A474–A478. 10.1149/2.0851503jes.

