# Supplementary material for: Toward Sustainable Li–S Battery Using Scalable Cathode and Safe Glyme-Based Electrolyte
Source: ACS Appl Energy Mater. 2023 Nov 8;6(22):11560–72. doi: 10.1021/acsaem.3c01966 (PMC10685327; doi:10.1021/acsaem.3c01966)
Supplement: Supplementary file 1 — ae3c01966_si_001.pdf [file ae3c01966_si_001.pdf]

Supporting Information

Toward Sustainable Li–S Battery Using Scalable Cathode and Safe Glyme-Based Electrolyte

Vittorio Marangon,[a,b‡] Edoardo Barcaro,[b‡] Eugenio Scaduti,[b] Filippo Adami,[b] Francesco Bonaccorso,[a,c] Vittorio Pellegrini,[a,c] Jusef Hassoun,[a,b,d] *

[a] *Graphene Labs, Istituto Italiano di Tecnologia, via Morego 30, Genoa, 16163, Italy*

[b] *Department of Chemical, Pharmaceutical and Agricultural Sciences, University of Ferrara, Via Fossato di Mortara 17, Ferrara, 44121, Italy*

[c] *BeDimensional S.p.A., Lungotorrente Secca 30R, Genova, 16163, Italy*

[d] *National Interuniversity Consortium of Materials Science and Technology (INSTM), University of Ferrara Research Unit, Via Fossato di Mortara, 17, 44121, Ferrara, Italy*

[‡] Authors equally contributed

* Corresponding author. E-mail addresses: jusef.hassoun@unife.it, jusef.hassoun@iit.it.

Pages number: 14

Figures number: 6

Tables number: 5

Figure S1 reports the Nyquist plots obtained via EIS at various temperatures (Fig. S1a-c) in SS|electrolyte|SS cells to calculate the ionic conductivity of the TE-5% (Fig. S1a), TE-10% (Fig. S1b) and TE-15% (Fig. S1c) electrolytes, as well as the chronoamperometric curves (Fig. S1d-f) alongside Nyquist plots (insets in Fig. S1d-f) recorded on Li|Li cells used to calculate the Li^+ transference number (t^+) of the TE-5% (Fig. S1d), TE-10% (Fig. S1e) and TE-15% (Fig. S1f) solutions through Bruce-Vincent-Evans method (see eq. 2 in the Manuscript).[1] The Nyquist plots are analyzed through NLLS fitting method using the Boukamp software[2,3] to obtain the electrolyte resistance (R_e) from the spectra of Figure S1a-c and the electrode/electrolyte interphase resistance (R_i) form the spectra of Figure S1d-f. See Experimental section in the Manuscript for details on NLLS method, Figure 1c for the ionic conductivity trends, Figure 1f for graphical representation of the t^+ values and Table 2 for additional parameters used in eq. 2.

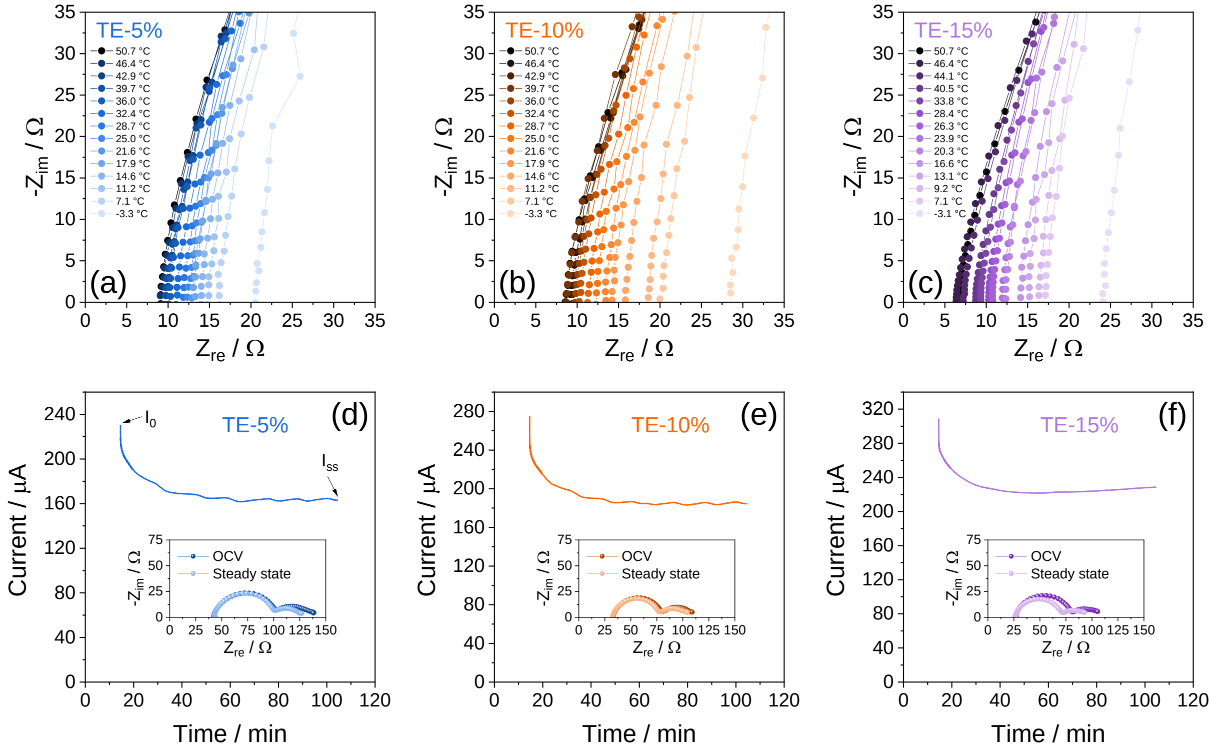

Figure S1. (a-c) Nyquist plots recorded by EIS at various temperatures on SS|electrolyte|SS cells using either **(a)** TE-5%, **(b)** TE-10% or **(c)** TE-15% to evaluate ionic conductivity; see respective conductivity plots in Figure 1c of the Manuscript; EIS frequency range: 500 kHz – 100 Hz; **(d-f)** chronoamperometric curves and Nyquist plots (insets) recorded to calculate the Li^+ transference number (t^+) for the **(d)** TE-5%, **(e)** TE-10% and **(f)** TE-15% electrolytes in Li|Li cell using the Bruce-Vincent-Evans method (eq. 2 in the Manuscript);[1] EIS frequency range: 500 kHz – 100 mHz; alternate voltage signal: 10 mV; chronoamperometry voltage: 30 mV; see Table 2 in the Manuscript for the parameters used to evaluate t^+ and Figure 1f for a histogram representation of the obtained values. See Table 1 in the Manuscript for electrolyte acronyms.

Figure S2 shows the Nyquist plots obtained through EIS measurements performed in the first 14 h (Fig. S2a-c) and upon 18 days (Fig. S2d-f) since the assembly of Li|Li cells using either TE-5% (Fig. S2a, d), TE-10% (Fig. S2b, e) or TE-15% (Fig. S2c, f). The NLLS analyses reported in Tables S1, S2 and S3, respectively, allowed the evaluation of R_i through cell aging,[2,3] revealing values between 85 and 154 Ω for TE-5%, between 84 and 129 Ω for TE-10% and between 50 and 86 Ω for TE-15%. The results suggest crucial role of the selected electrolyte in stabilizing the SEI on lithium surface and mitigate the resistance of Li^+ transport. See Experimental section in the Manuscript for details on NLLS method, and Figure 1c for the ionic conductivity trends, and Figure 2a for the resistance trends.

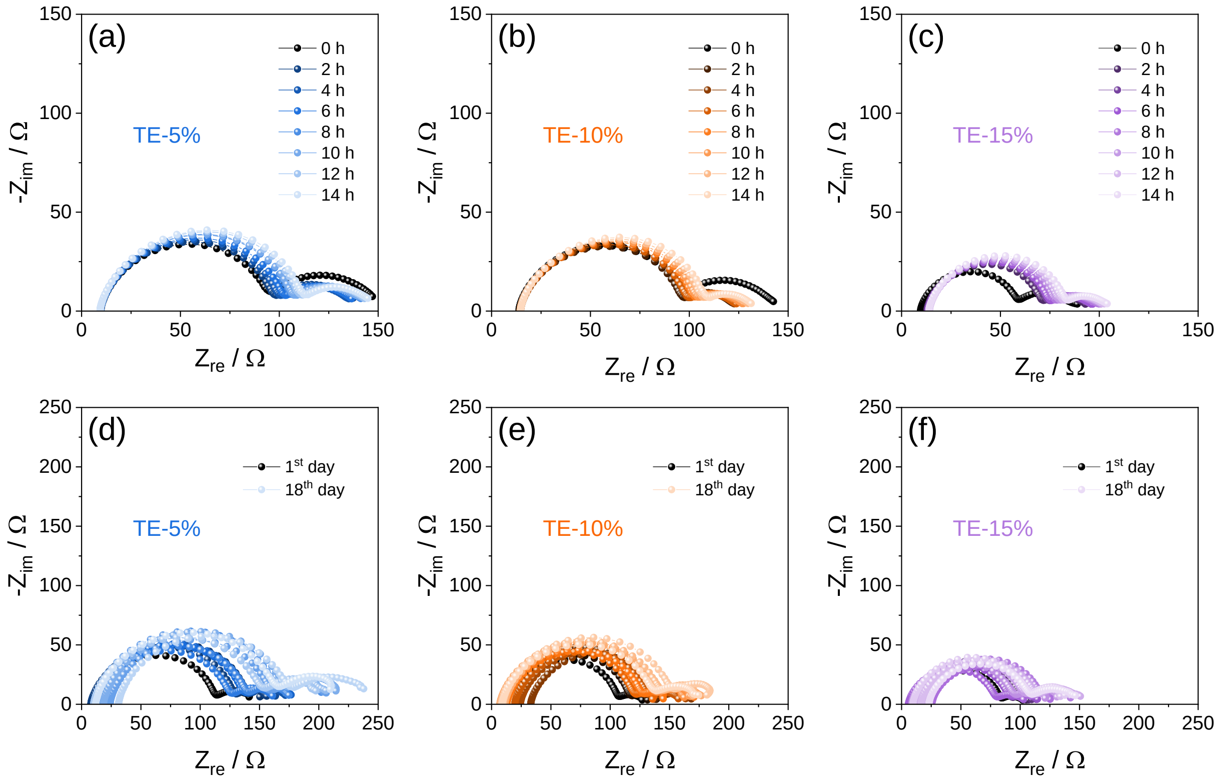

Figure S2. Nyquist plots achieved through EIS upon aging of Li|Li cells using either **(a, d)** TE-5%. **(b, e)** TE-10% or **(c, f)** TE-15%. The Nyquist plots are analyzed through NLLS method[2,3] and the results are reported in Tables S1, S2 and S3, while the corresponding interphase resistance trends are displayed in Figure 2a in the Manuscript. Frequency range: 500 kHz – 100 mHz; alternate voltage signal: 10 mV. See Table 1 in the Manuscript for electrolyte acronyms.

Cell condition	Circuit	R_i [Ω]	χ^2
After assembly	$R_e(R_iQ_i)(R_wQ_w)$	85.2 ± 0.7	1×10^{-4}
2 hours	$R_e(R_iQ_i)(R_wQ_w)$	89.4 ± 0.6	1×10^{-4}
4 hours	$R_e(R_iQ_i)(R_wQ_w)$	92.2 ± 0.5	1×10^{-4}
6 hours	$R_e(R_iQ_i)(R_wQ_w)$	94.6 ± 0.6	1×10^{-4}
8 hours	$R_e(R_iQ_i)(R_wQ_w)$	98.8 ± 0.6	1×10^{-4}
10 hours	$R_e(R_iQ_i)(R_wQ_w)$	100 ± 1	1×10^{-4}
12 hours	$R_e(R_iQ_i)(R_wQ_w)$	103 ± 1	1×10^{-4}
14 hours	$R_e(R_iQ_i)(R_wQ_w)$	105 ± 1	2×10^{-4}
1 day	$R_e(R_iQ_i)(R_wQ_w)$	106 ± 1	2×10^{-4}
2 days	$R_e(R_iQ_i)(R_wQ_w)$	130 ± 1	2×10^{-4}
3 days	$R_e(R_iQ_i)(R_wQ_w)$	123 ± 1	3×10^{-4}
4 days	$R_e(R_iQ_i)(R_wQ_w)$	128 ± 1	3×10^{-4}
5 days	$R_e(R_iQ_i)(R_wQ_w)$	127 ± 1	2×10^{-4}
6 days	$R_e(R_iQ_i)(R_wQ_w)$	130 ± 1	1×10^{-4}
7 days	$R_e(R_iQ_i)(R_wQ_w)$	129 ± 1	1×10^{-4}
8 days	$R_e(R_iQ_i)(R_wQ_w)$	116 ± 1	8×10^{-5}
9 days	$R_e(R_iQ_i)(R_wQ_w)$	116 ± 1	7×10^{-5}
10 days	$R_e(R_iQ_i)(R_wQ_w)$	157 ± 1	2×10^{-4}
11 days	$R_e(R_iQ_i)(R_wQ_w)$	138 ± 1	8×10^{-5}
14 days	$R_e(R_iQ_i)(R_wQ_w)$	156 ± 1	2×10^{-4}
15 days	$R_e(R_iQ_i)(R_wQ_w)$	143 ± 1	1×10^{-4}
16 days	$R_e(R_iQ_i)(R_wQ_w)$	143 ± 1	2×10^{-4}
17 days	$R_e(R_iQ_i)(R_wQ_w)$	146 ± 1	4×10^{-5}
18 days	$R_e(R_iQ_i)(R_wQ_w)$	154 ± 1	1×10^{-4}

Table S1. NLLS analyses[2,3] performed on the Nyquist plots displayed in Figure S2a and d recorded by EIS upon aging of a Li|TE-5%|Li cell; frequency range: 500 kHz – 100 mHz; alternate voltage signal: 10 mV. See Figure 2a in the Manuscript for corresponding trend and Table 1 for electrolyte acronyms.

Cell condition	Circuit	R_1 [Ω]	χ^2
After assembly	$R_e(R_iQ_i)(R_wQ_w)$	83.5 ± 0.8	2×10^{-4}
2 hours	$R_e(R_iQ_i)(R_wQ_w)$	83.9 ± 0.8	2×10^{-4}
4 hours	$R_e(R_iQ_i)(R_wQ_w)$	86.1 ± 0.8	2×10^{-4}
6 hours	$R_e(R_iQ_i)(R_wQ_w)$	86.9 ± 0.7	2×10^{-4}
8 hours	$R_e(R_iQ_i)(R_wQ_w)$	90.4 ± 0.8	2×10^{-4}
10 hours	$R_e(R_iQ_i)(R_wQ_w)$	92.0 ± 0.9	2×10^{-4}
12 hours	$R_e(R_iQ_i)(R_wQ_w)$	94.5 ± 0.7	1×10^{-4}
14 hours	$R_e(R_iQ_i)(R_wQ_w)$	96.6 ± 0.7	1×10^{-4}
1 day	$R_e(R_iQ_i)(R_wQ_w)$	97.1 ± 0.9	3×10^{-4}
2 days	$R_e(R_iQ_i)(R_wQ_w)$	108 ± 1	2×10^{-4}
3 days	$R_e(R_iQ_i)(R_wQ_w)$	121 ± 1	3×10^{-4}
4 days	$R_e(R_iQ_i)(R_wQ_w)$	125 ± 1	2×10^{-4}
5 days	$R_e(R_iQ_i)(R_wQ_w)$	114 ± 1	9×10^{-5}
6 days	$R_e(R_iQ_i)(R_wQ_w)$	117 ± 1	8×10^{-5}
7 days	$R_e(R_iQ_i)(R_wQ_w)$	115 ± 1	1×10^{-4}
8 days	$R_e(R_iQ_i)(R_wQ_w)$	114 ± 1	2×10^{-4}
9 days	$R_e(R_iQ_i)(R_wQ_w)$	118 ± 1	1×10^{-4}
10 days	$R_e(R_iQ_i)(R_wQ_w)$	128 ± 1	2×10^{-4}
11 days	$R_e(R_iQ_i)(R_wQ_w)$	130 ± 1	3×10^{-4}
14 days	$R_e(R_iQ_i)(R_wQ_w)$	134 ± 2	3×10^{-4}
15 days	$R_e(R_iQ_i)(R_wQ_w)$	132 ± 1	1×10^{-4}
16 days	$R_e(R_iQ_i)(R_wQ_w)$	137 ± 1	3×10^{-4}
17 days	$R_e(R_iQ_i)(R_wQ_w)$	145 ± 1	2×10^{-4}
18 days	$R_e(R_iQ_i)(R_wQ_w)$	129 ± 1	2×10^{-4}

Table S2. NLLS analyses[2,3] performed on the Nyquist plots displayed in Figure S2b and e recorded by EIS upon aging of a Li|TE-10%|Li cell; frequency range: 500 kHz – 100 mHz; alternate voltage signal: 10 mV. See Figure 2a in the Manuscript for corresponding trend and Table 1 for electrolyte acronyms.

Cell condition	Circuit	R_1 [Ω]	χ^2
After assembly	$R_e(R_iQ_i)(R_wQ_w)$	49.8 ± 0.3	6×10^{-5}
2 hours	$R_e(R_iQ_i)(R_wQ_w)$	59.5 ± 0.4	1×10^{-4}
4 hours	$R_e(R_iQ_i)(R_wQ_w)$	62.3 ± 0.4	8×10^{-5}
6 hours	$R_e(R_iQ_i)(R_wQ_w)$	63.0 ± 0.4	8×10^{-5}
8 hours	$R_e(R_iQ_i)(R_wQ_w)$	65.5 ± 0.4	7×10^{-5}
10 hours	$R_e(R_iQ_i)(R_wQ_w)$	67.1 ± 0.4	8×10^{-5}
12 hours	$R_e(R_iQ_i)(R_wQ_w)$	69.0 ± 0.4	9×10^{-5}
14 hours	$R_e(R_iQ_i)(R_wQ_w)$	71.1 ± 0.5	1×10^{-4}
1 day	$R_e(R_iQ_i)(R_wQ_w)$	72.1 ± 0.4	7×10^{-5}
2 days	$R_e(R_iQ_i)(R_wQ_w)$	83.0 ± 0.6	1×10^{-4}
3 days	$R_e(R_iQ_i)(R_wQ_w)$	91.4 ± 0.9	2×10^{-4}
4 days	$R_e(R_iQ_i)(R_wQ_w)$	87.4 ± 0.8	2×10^{-4}
5 days	$R_e(R_iQ_i)(R_wQ_w)$	82.9 ± 0.6	1×10^{-4}
6 days	$R_e(R_iQ_i)(R_wQ_w)$	84.2 ± 0.4	6×10^{-5}
7 days	$R_e(R_iQ_i)(R_wQ_w)$	81.3 ± 0.8	2×10^{-4}
8 days	$R_e(R_iQ_i)(R_wQ_w)$	74.5 ± 0.4	8×10^{-5}
9 days	$R_e(R_iQ_i)(R_wQ_w)$	82.6 ± 0.9	9×10^{-5}
10 days	$R_e(R_iQ_i)(R_wQ_w)$	95.9 ± 0.7	7×10^{-5}
11 days	$R_e(R_iQ_i)(R_wQ_w)$	86.8 ± 0.8	2×10^{-4}
14 days	$R_e(R_iQ_i)(R_wQ_w)$	93.1 ± 0.9	1×10^{-4}
15 days	$R_e(R_iQ_i)(R_wQ_w)$	93.8 ± 0.5	5×10^{-5}
16 days	$R_e(R_iQ_i)(R_wQ_w)$	92.1 ± 0.7	8×10^{-5}
17 days	$R_e(R_iQ_i)(R_wQ_w)$	98.0 ± 0.8	1×10^{-4}
18 days	$R_e(R_iQ_i)(R_wQ_w)$	85.9 ± 0.5	3×10^{-5}

Table S3. NLLS analyses[2,3] performed on the Nyquist plots displayed in Figure S2c and f recorded by EIS upon aging of a Li|TE-15%|Li cell; frequency range: 500 kHz – 100 mHz; alternate voltage signal: 10 mV. See Figure 2a in the Manuscript for corresponding trend and Table 1 for electrolyte acronyms.

Figure S3 displays higher magnification for the anodic stability LSV curves (Fig. S3a, see Figure 2b-d in the Manuscript) and Li stripping-deposition tests (Fig. S3b-d, see Figure 2e in the Manuscript). The raise of DOL concentration causes the expected decrease in anodic stability due to the DOL-ring cleavage on Li surface promoting SEI formation (Fig. S3a), while, at the same time, lowers the polarization as likely ascribed to viscosity reduction of the TEGDME-based electrolyte (Fig. S3b-d).

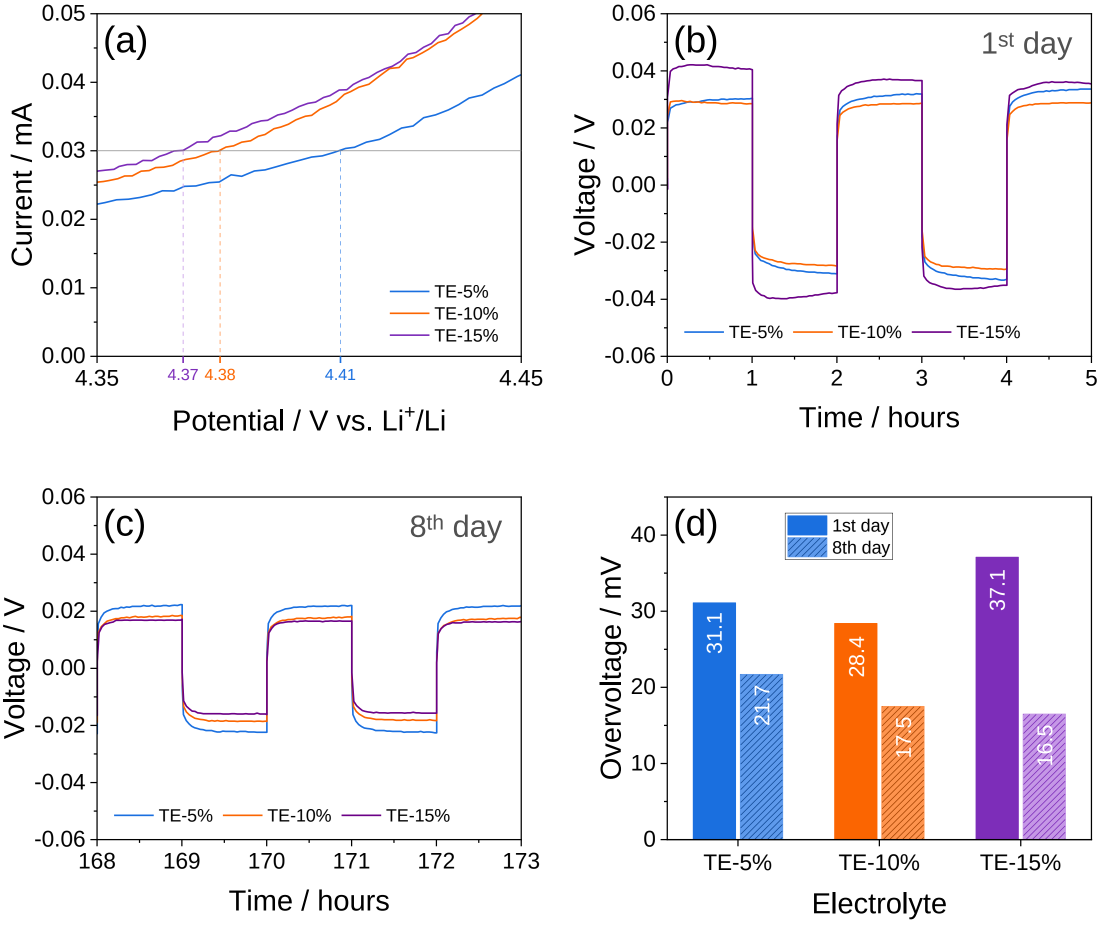

Figure S3. Magnifications of **(a)** anodic stability curves determined in Figure 2b-d of the Manuscript by LSV and **(b, c)** lithium-stripping deposition tests performed on Li|Li cells displayed in Figure 2e of the Manuscript at the **(b)** 1st day and **(c)** 8th day for the TE-5%, TE-10% and TE-15% electrolytes; **(d)** histogram representation of the overvoltage polarization related to the lithium-stripping deposition tests at the 1st and 8th day of measurement. LSV range: from OCV to 5.0 V vs. Li^+/Li; scan rate: 0.1 mV s^{-1}. See Table 1 in the Manuscript for electrolyte acronyms.

Table S4 compares the chemical-physical properties of TE-5%, TE-10% and TE-15% with other electrolytes used in Li-S battery according to previous literature reported in the Manuscript. The results displayed in this work demonstrate the achievement of a compromise between DOL:DME and TEGDME-based solutions for the properties of TE-5%, TE-10% and TE-15%.

**Electrolyte**	**Thermal stability [°C]**	**Conductivity at room temperature [S cm^{-1}]**	**Li^+ transference number**	**Anodic stability [V vs. Li^+/Li**	**Reference in the Manuscript**
TE-5%	150 -200	1.5×10^{-3}	0.55	4.41	This work
TE-10%	150-200	1.5×10^{-3}	0.53	4.38	
TE-15%	150-200	1.5×10^{-3}	0.50	4.37	
DOL:DME solution	50	2×10^{-3}	0.67	4.0	53, 18
DEGDME solution	70	2×10^{-3}	0.50	4.6	13
TEGDME solution	200	1×10^{-3}	0.49	4.2	19
PEGDME (M_w = 500 g mol^{-1}) solution	350	4×10^{-4}	0.44	>4.5	18

Table S4. Comparison of the physical-chemical properties of TE-5%, TE-10% and TE-15% with other electrolytes employed in Li-S cell according to previous literature papers reported in the Manuscript. See Table 1 in the Manuscript for electrolyte acronyms.

Figure S4 shows the galvanostatic voltage profiles related to Li-S cells using the S:MWCNTs 90:10 *w/w* electrode and the DOL:DME-control electrolyte cycled either at C/5 (Fig. S4a) or C/3 (Fig. S4b). Both the cells exhibit reversible conversion of Li and S to Li_2S_x species during discharge through two plateaus between 1.9 and 2.3 V and their reverse oxidation during charge between 2.3 and 2.5 V, leading to a cycle life of 200 cycles. The respective cycling trends in Figure 4 in the Manuscript reveal satisfactory capacity retention and Coulombic efficiency approaching 100% for the whole test.

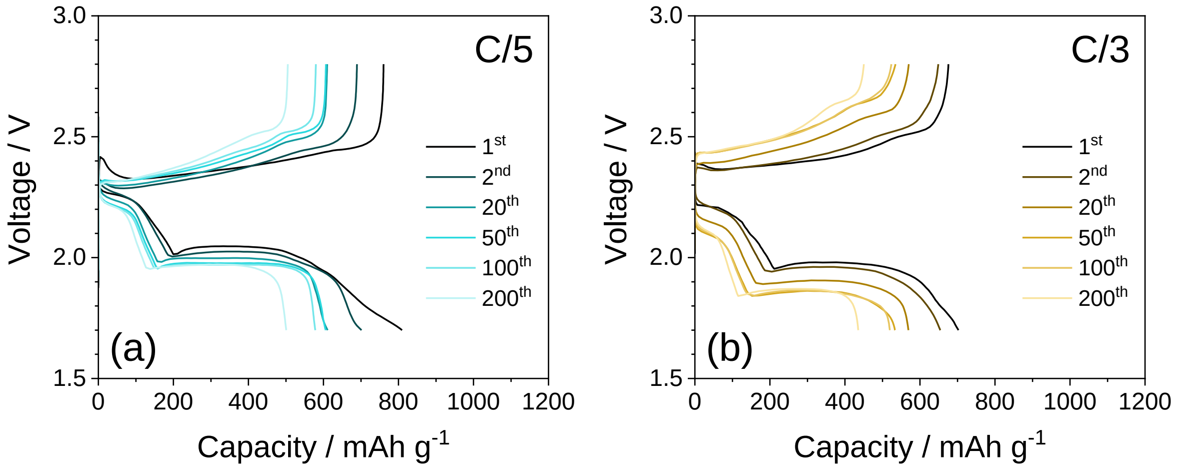

Figure S4. Voltage profiles of Li cells using the DOL:DME-control electrolyte coupled with the S:MWCNTs 90:10 *w/w* electrode tested at either **(a)** C/5 or **(b)** C/3 between 1.7 and 2.8 V. E/S ratio: 10 μL mg^{-1}. See corresponding capacity and Coulombic efficiency trends in Figure 4 of the Manuscript.

Figure S5 displays the voltammograms obtained by a Li cell using the DOL:DME-control electrolyte and the S:MWCNTs 90:10 *w/w* electrode. The potential profiles confirm the remarkable reversibility and stability of the Li-S conversion process, evidencing two reduction steps at 2.25 and 1.95 V vs. Li^+/Li reversed in a double-charge wave at 2.5 V vs. Li^+/Li at the first cycle and well-overlapped curves during subsequent scans.

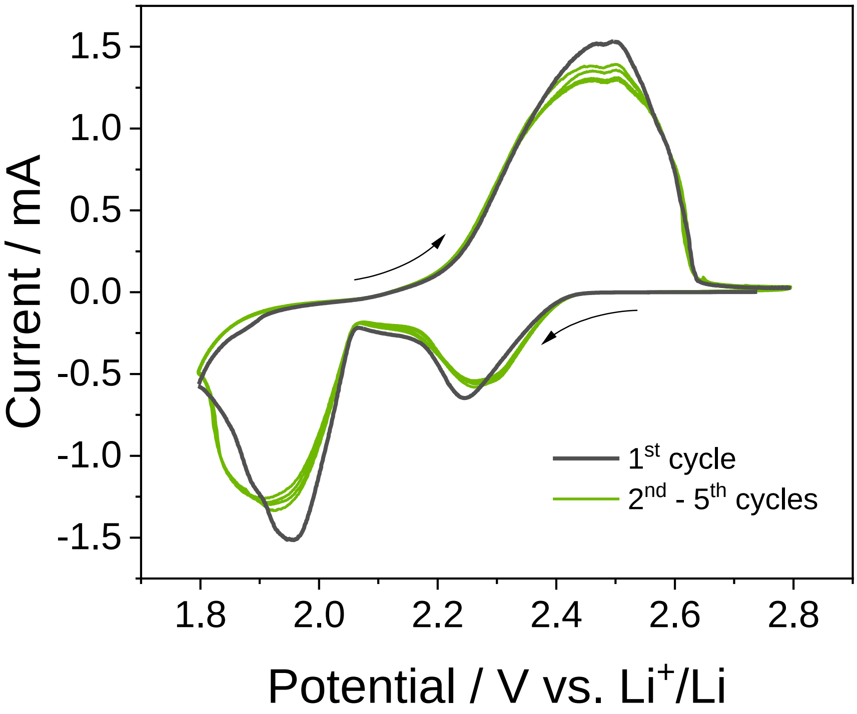

Figure S5. CV test performed on Li cell using the DOL:DME-control electrolyte coupled with the S:MWCNTs 90:10 *w/w* electrode. Potential range: 1.8 – 2.8 V vs. Li^+/Li. Scan rate: 0.1 mV s^{-1}.

Table S5 reports a comparison of the results achieved in this work with literature papers reported in the Manuscript where glyme-based electrolytes are coupled with a sulfur electrode in Li-S batteries. The comparison is carried out by taking in consideration operative conditions similar to those adopted herein, that is, an electrolyte without addition of lithium polysulfides (Li_2S_x) and with aluminum-based current collector. The outcomes show that the performance achieved by our Li-S batteries are in line with previous works, with additional bonus of enhanced sulfur loading and the safety content.

**Electrolyte**	**E/S ratio [µL mg^{-1}]**	**Sulfur in composite [wt.%]**	**Electrode sulfur loading [mg cm^{-2}]**	**C-rate (1C = 1675 mA g^{-1})**	**Steady state capacity [mAh g^{-1}]**	**Reference in the Manuscript**
TE-10%	15	90	2.1	C/5	500	This work
DOL:DME, LiTFSI, $LiNO_3$	15	70	1.5 – 2.0	C/5	790	25
DEGDME, LiTFSI	5	70	2.0	C/5	800	12
TREGDME, LiTFSI	/	60	0.5	C/18	700	14
TEGDME, TFTFE, LiTFSI	18	80	1.2	C/3	550	16

Table S5. Comparison of the electrochemical performance obtained from the Li-S cell using TE-10% (see Figure 5 in the Manuscript for voltage profiles and cycling trend) with previous literature works reported in the Manuscript. See Table 1 in the Manuscript for electrolyte acronym.

Figure S6 shows the rate capability tests performed on Li-S cells using the TE-5%, TE-10% and TE-15% electrolytes. The voltage profiles (Fig. S6a-c) show a satisfactory performance from C/20 to C/8 for all the solutions, while only TE-10% and TE-15% display suitable cycling at C/5. In addition, all the solutions present limited response at C/3, and almost total deactivation of the conversion process at C/2. The corresponding cycling trend (Fig. S6d) further evidences the superior rate capability of TE-15%, which shows stable cycling at C/5 with steady state capacity of 640 mAh g^{-1}, followed by TE-10% delivering 500 mAh g^{-1} despite a decreasing trend.

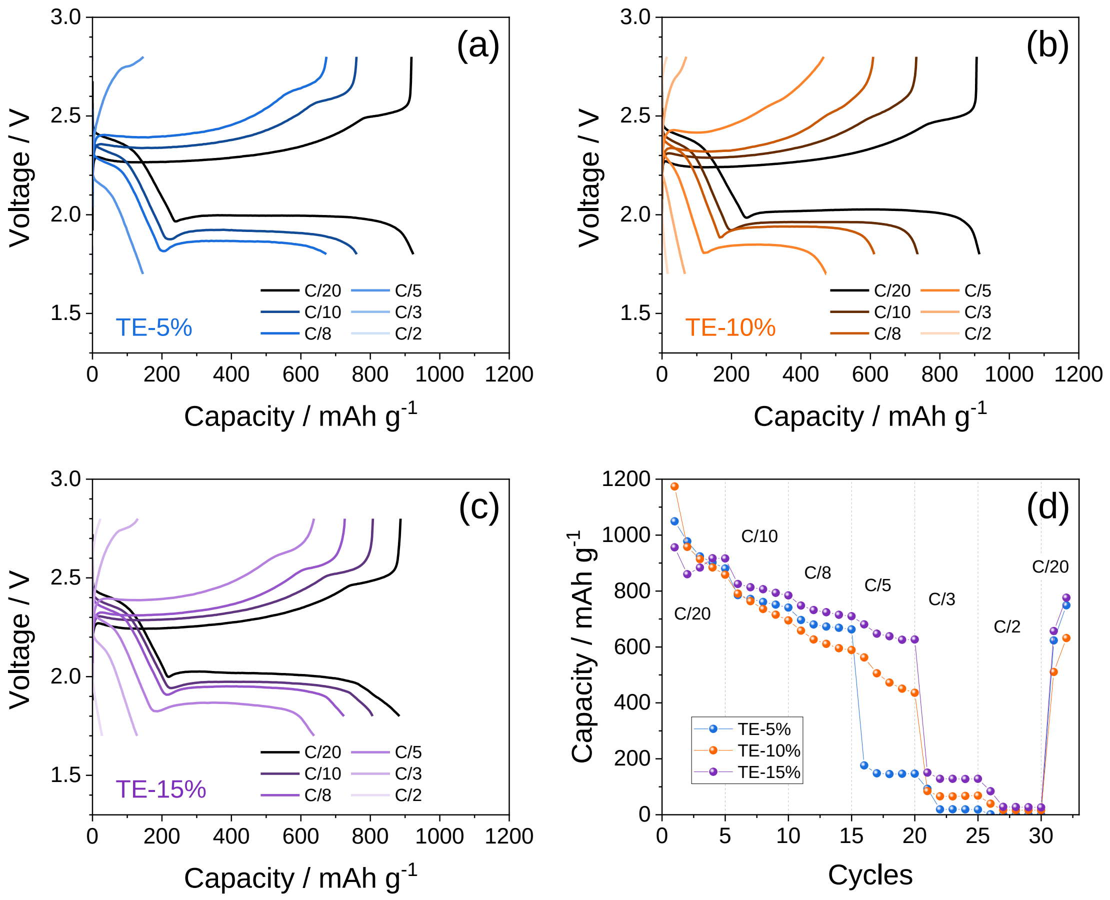

Figure S6. (a-c) Voltage profiles and **(d)** cycling trend related to rate capability tests of Li-S cells using either **(a)** TE-5%, **(b)** TE-10% or **(c)** TE-15%. Current rate increased every 5 cycles from C/20 to C/10, C/8, C/5, C/3, C/2 and decreased back to C/20 after 30 cycles. Voltage ranges: 1.8 – 2.8 V from C/20 to C/8, 1.7 – 2.8 V from C/5 to C/2. E/S ratio: 15 μL mg^{-1}. Sulfur loading: 2 mg cm^{-2}. See Table 1 in the Manuscript for electrolyte acronyms.

References

1 J. Evans, C. A. Vincent and P. G. Bruce, *Polymer (Guildf)*, 1987, **28**, 2324–2328.

2 B. Boukamp, *Solid State Ion*, 1986, **18–19**, 136–140.

3 B. Boukamp, *Solid State Ion*, 1986, **20**, 31–44.
